# Influence of cement soil column on soil behavior

**DOI:** 10.1038/s41598-025-31306-5

**Published:** 2025-12-29

**Authors:** Abd E. L. Samee W. Nashaat, A. M. Kamar

**Affiliations:** 1 Civil Engineering Dep., Faculty of Engineering, Beni- Suef University, Beni- Suef, Egypt; 2 Faculty of Engineering, Sinai University, El Arish, Egypt

**Keywords:** Cement-soil, Bearing capacity, Settlement, Improvement, PLAXIS 3D, Engineering, Environmental sciences, Materials science

## Abstract

The present study is mainly based on the determination of the effect of cement soil on improving the bearing capacity of weak soil and decreasing the settlement. The program consists of installing a cement soil column with a fixed diameter (D = 0.1 m), various percentages of cement (0 ,3, 6, 9 and 12%) of soil weight and fixed length (L = 1.0 m). The column is supported by a circular steel plate with a diameter of 0.45 m and thickness 0.25 m to ensure the load is distributed uniformly from the hydraulic jack to the cement soil column in a very fine-sandy soil with traces of clay in El-Arish region placed in a soil chamber, and subjected to compressive axial load. The displacement underneath the plate was measured with a 2- dial gauge. Finite element package of a PLAXIS 3D version 2021 (A finite element code for soil analysis) has been done for the experimental program to compare the theoretical and experimental results. The obtained experimental test results indicated that increasing the percentage of cement has a significant effect on decreasing the settlement of soil and enhancing the bearing capacity of soil. As the untreated soil exhibited high compressibility, reaching a settlement of 1.65 cm under a stress of 4 kg/cm^2^. This was reduced to 0.9 cm with 12% cement (demonstrating 45% reduction in settlement). Also, Study the effect of increasing number of cement column in the same area theoretically where (N = 1,2,3and4) cement column on settlement. On the other hand, studying the effect of spacing between cement soil column for 4 cement columns with various spacing S = (2.5D,3D,3.5D,4D) has been done theoretically. It was found that the settlement can be reduced by increasing the number of columns in a group but is adversely affected by increasing the spacing between them.

## Introduction

The stability and strength of subgrade soil are critical for the longevity and performance of overlying structures and pavements. In regions where subgrade soils are weak, soil improvement becomes a necessary engineering intervention. While traditional solutions like deep foundations exist, ground improvement with chemical stabilizers such as lime, cement, and bitumen offers a more economical and sustainable alternative for many projects by enhancing key properties like shear strength, compressibility, and bearing capacity. Extensive research has been conducted on soil stabilization. The effectiveness of lime in treating soft, organic clays is well-established, with studies like Mohamed A. Sakr, et al.^1^ demonstrating its success even in soils with high organic content. Beyond pure chemical additives, research has evolved to include reinforced systems, such as the fiber-reinforced lime columns studied by Nagy A. El Mahallawy, Ahmad S. Rashed^2^, which showed significant settlement reduction. H.Ghobadi Y. Abdilor R. Babazadeh^3^ confirmed that specific clay types can be effectively stabilized with lime additions around 7%. Armin Roohbakhshan1, Behzad Kalantari^4^ investigated the effectiveness of using rock dust and lime to stabilize fine-grained clay (CL) in the laboratory. Furthermore, comparative studies by researchers like Akiije^5^ and Agashua, Lucia. O; Ogbiye, Adebanji. S^6^ have highlighted that the performance of stabilizers like cement, lime, and bitumen is highly dependent on the parent soil’s mineralogy, with cement often showing superior strength gains but at a higher cost and environmental impact. Shahzada Omer Manzoor and Aadil Yousuf^7^ discussed the importance of lime stabilization through lime-columns, lime treatment of pavement layers. The impact of lime treatment was found to increase soil strength. Christopher Ehizemhen Igibah^8^ showed that the addition of hydrated lime and Portland cement had positive effects on enhancing the strength of inferior or unstable soils. This has spurred recent interest in reducing cement usage through additives, as explored by Dao Phu-Yen^9^. similarity Chen et al.^10^ study a comprehensive analysis of the load–displacement behavior of soil–cement columns. However, a direct and systematic comparison of the mechanical and durability performance of these stabilizers lime, cement, and bitumen on the very fine-sandy soil with traces of clay in El-Arish region is absent from the literature. This gap makes it challenging for engineers in this region to select the most technically sound and cost-effective stabilization strategy. The main objectives of this study are to: (1) Characterize the geotechnical properties of the natural of very fine-sandy soil with traces of clay in El-Arish region (2) Determine the strength and compressibility improvements achieved by adding 3%, 6%, 9%, and 12% of cement. (3) Study the effect of increasing number of cement column in the same area theoretically where (N = 1,2,3 and 4) cement column on settlement.

## Experimental program

The experimental program was conducted to study the effect of adding of cement soil column on improving the bearing capacity of weak soil and decrease the settlement of it**.** In this experimental program Five models were used in this program. First model studying the soil without adding any percentage of cement, and the other four models studying the soil by adding cement column with various percentage of cement. Their length was constant (1.0 m) and diameters (0.1 m) with various percentage of cement (3,6,9 and 12%). The columns were tested in a set up under axial load. displacement was measured simultaneously. The program consisted of installing test cement soil columns in a very fine-sandy soil with traces of clay in El-Arish region, placing it in a soil chamber subjected to axial load. However, the testing was carried on one cement soil column have different percentage of cement and subjected to vertical load at centroid. The test program carried out was as shown in Table 1.

**Table 1:** Investigated experimental program.

No	Number of CS column	Length of CS column	CS column Diameter	Cement percentage
1	1	1.0 m	0.1 m	0%
2				3%
3				6%
4				9%
5				12%

Where:

CS: cement soil.

### Testing program

**Fig. 1:**
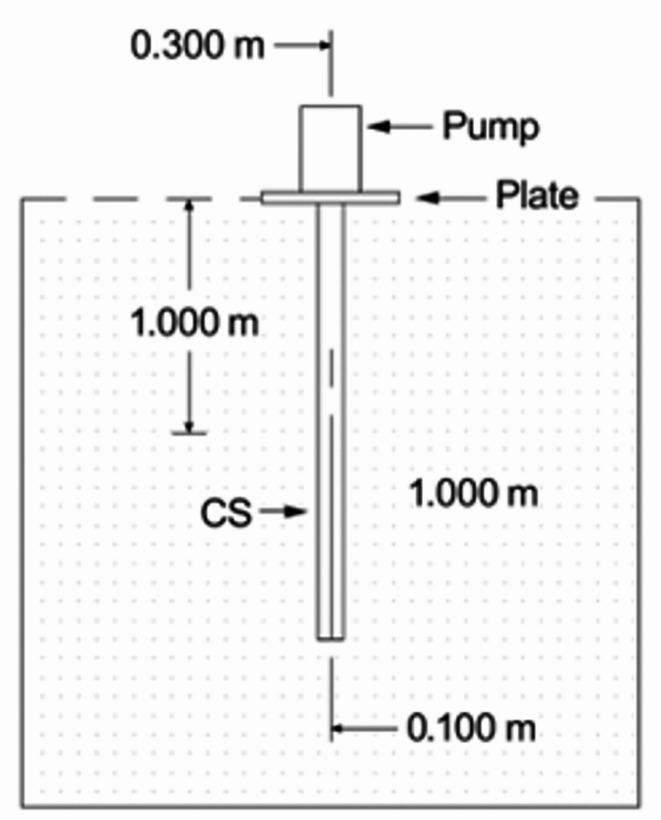
Cement column model with cap subjected to axial load.

The program consists of one cement soil column, height of them (L = 1.0 m) and diameters are constant (D = 0.1 m) and the cement soil column supported a circular steel plate with diameter = 0.45 m and thickness 25 mm and to ensure the load distributed from the hydraulic jack to the column and soil. The Cement columns have different percentage of cement (3,6,9 and 12%) of the weight of soil and subjected to vertical load at centroid as shown in Fig. 1.

#### Soil properties

**Fig. 2:**
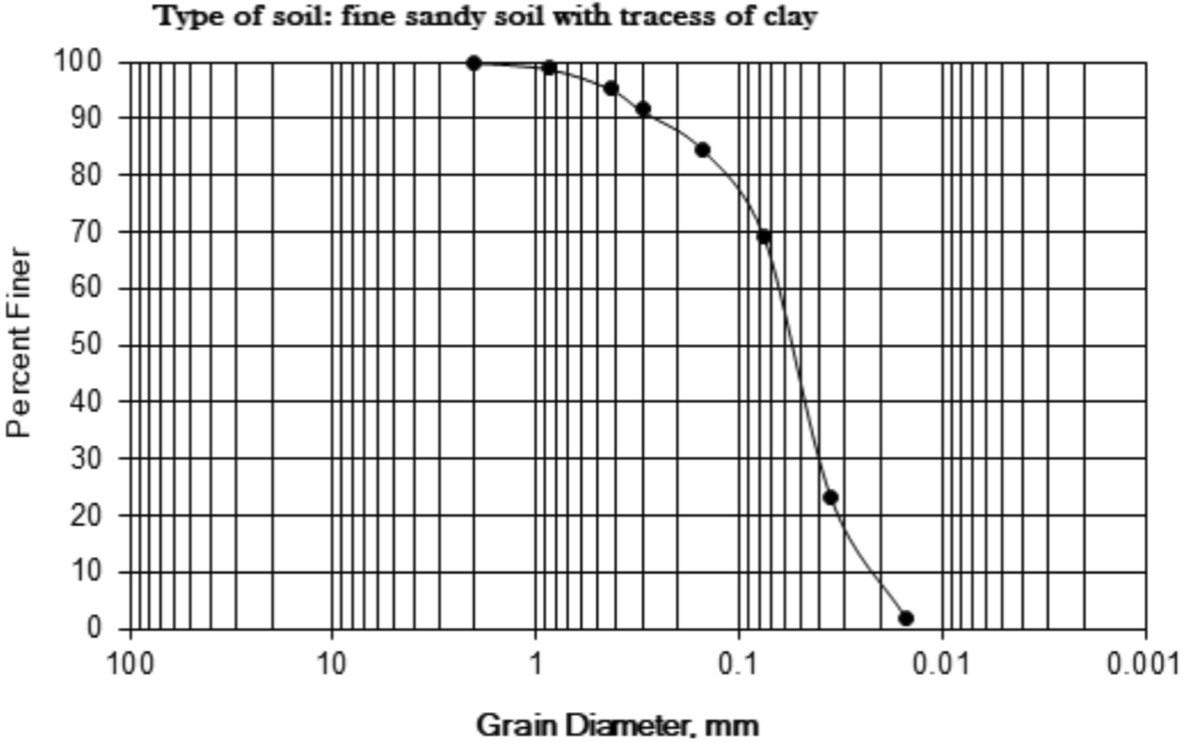
Grain size distribution.

**Fig. 3:**
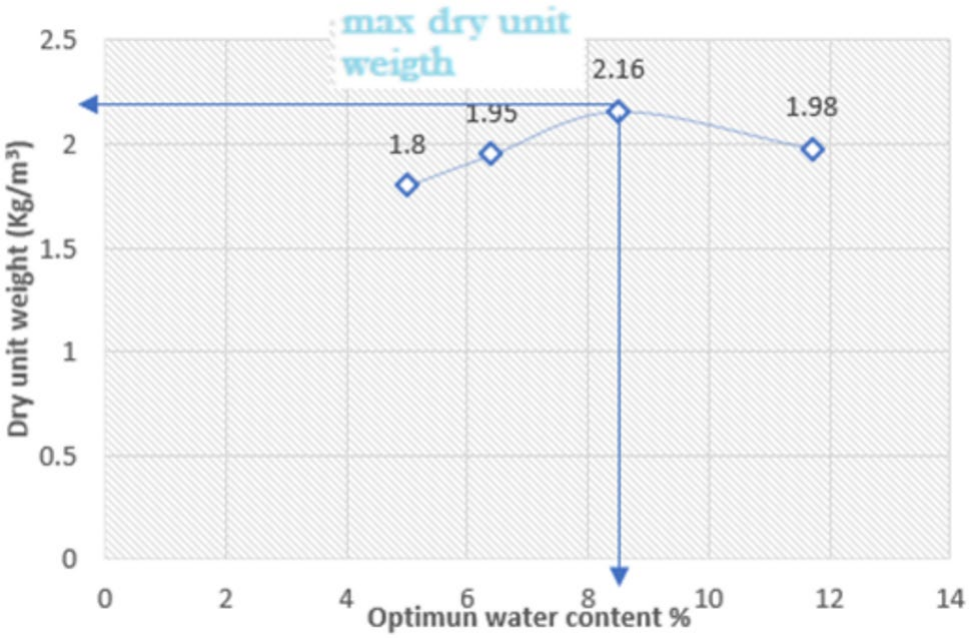
Relation between moisture content and dry unit weigh.

**Fig. 4:**
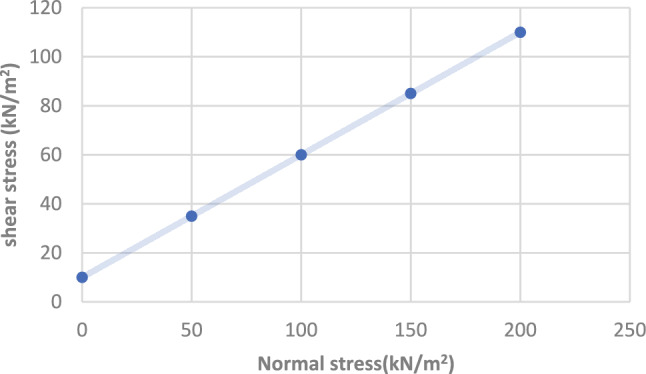
Relation between shear and Normal stress for direct shear box results of very fine sandy soil with traces of clay.

Important parameters for the predictive effort are the density and internal friction angle of the soil which have been calculated. The materials properties for the used soil layers were selected as shown in Table 2, Fig. 2 and Fig. 4. Modified proctor test was carried out on the sample as shown in Fig. 3.

**Table 2:** Properties for soil layers.

Parameters	Name	sandy soil with traces of clay	unit
unsaturated soil weight	ɣ_unsat_	18.25	kN/m^3^
saturated soil weight	ɣ_sat_	17	kN/m^3^
Poisson ratio	ʋ	0.25	-
Cohesion	ϲ	10	KN/m^2^
Friction angle	Ø	28	°

#### Used materials

The followings are details of materials used in cement column:

- a. Graded sand has been used as fine aggregate.
- b. Coarse aggregate used in the concrete mix is crushed stone.
- c. Clean fresh water free from is used for mixing column concrete.
- d. Portland Cement BS EN 197–1-CEM 42.4N is used in concrete for the all-experimental work (Fig. 4).

#### Cement column casting

**Fig. 5:**
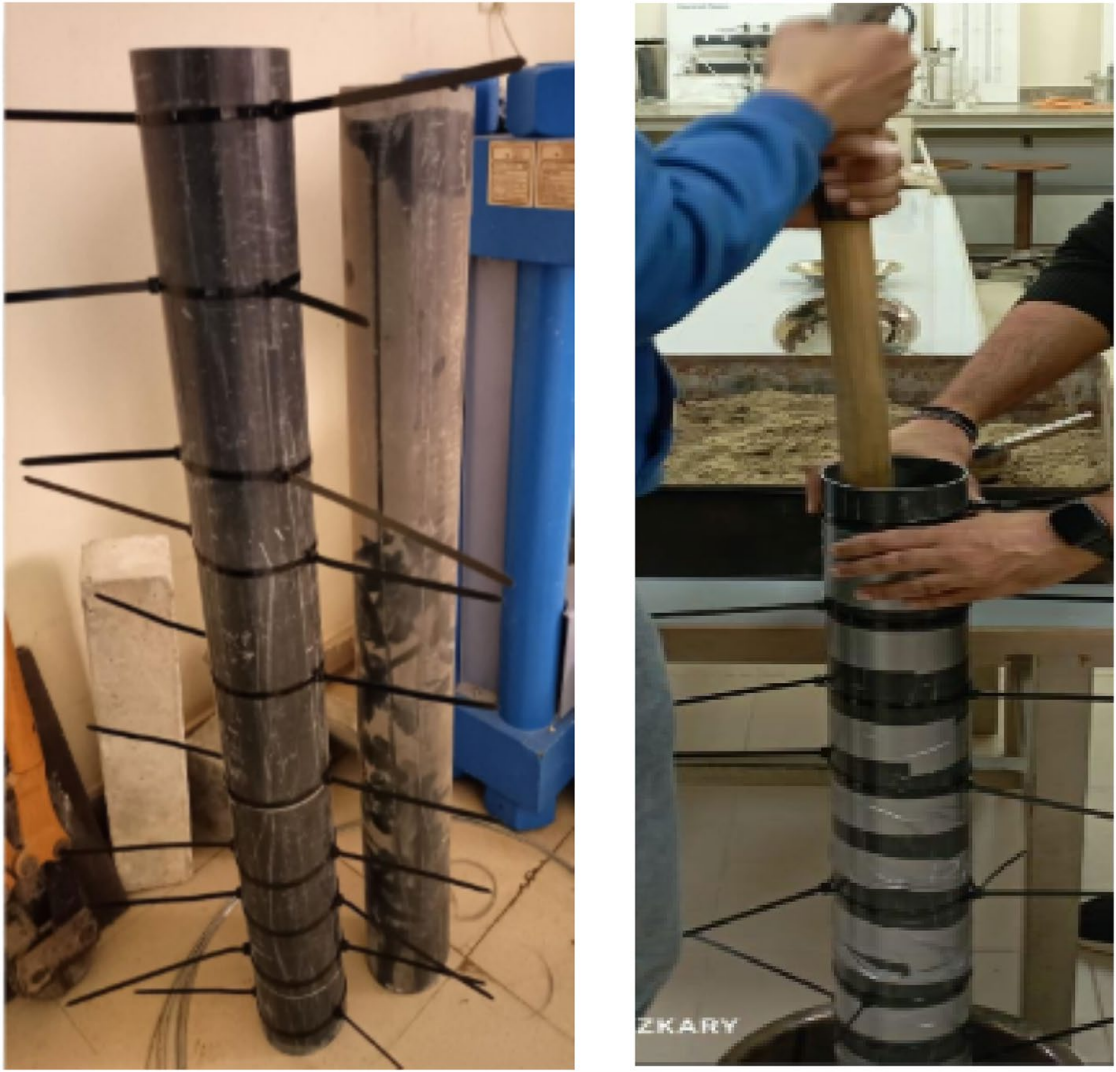
Description of forms and Casting of cement column.

A mechanical vibrator was used. All cylindrical columns were cured. The forms were made from plastic pipe where; the height and diameter of the column are fixed. The height is (L = 1 m) and the diameter is (Dia. = 0.1 m).and they are designed to enable casting the cement soil columns body, and then these columns are moved to setup location for testing. The details of the used form are shown in Fig. 5.

### Testing setup and procedure

The program tests were divided into two groups.

[1] First group: the soil was tested to determine its bearing capacity without any modification to the soil was axially loaded.

[2] Second group: the soil was tested by plate load test to determine its bearing capacity by carrying out four cement soil columns with different cement percentages of 3%, 6%, 9%, and 12% of sand weight were axially loaded by the followings:

i. The load is applied through the hydraulic jack and increased gradually.
ii. The increment is generally one-fifth of the expected safe bearing capacity or one-tenth of the ultimate bearing capacity.
iii. The applied load is noted from the pressure gauge.
iv. The settlement is observed for each increment and from dial gauge. After increasing the load-settlement should be observed after 1, 4, 10, 20, 40, and 60 min and then at hourly intervals until the rate of settlement is less than 0.02 mm per hour. The readings are noted.
v. After completing the collection of data for a particular loading, the next load increment is applied and readings are noted under new load.
vi. This increment and data collection is repeated until the maximum load is applied. The maximum load is generally 1.5 times the expected ultimate load or 3 times of the expected allowable bearing pressure.

#### Loading frame

**Fig. 6:**
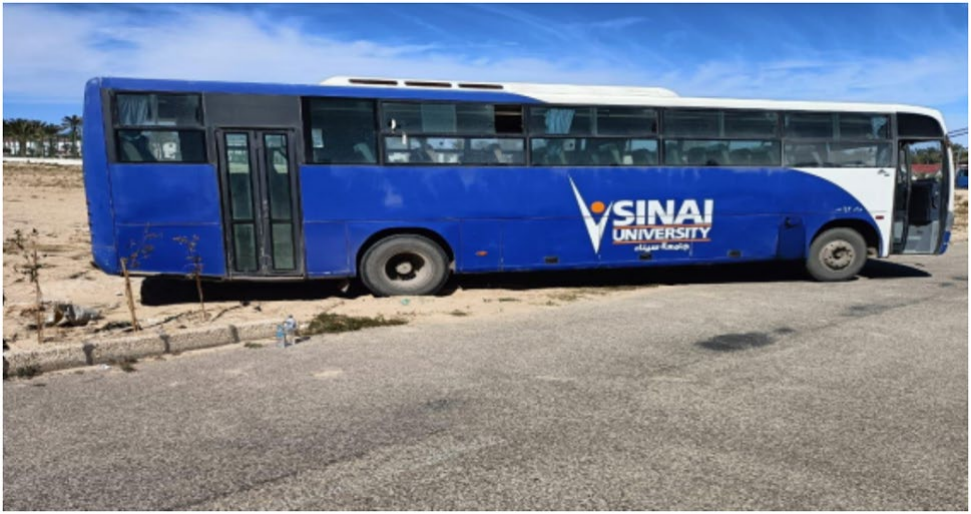
Loading frame.

We used the university bus to resist the expected maximum loads that might occur during the test, as shown in Fig. 6.

#### Loading jack

**Fig. 7:**
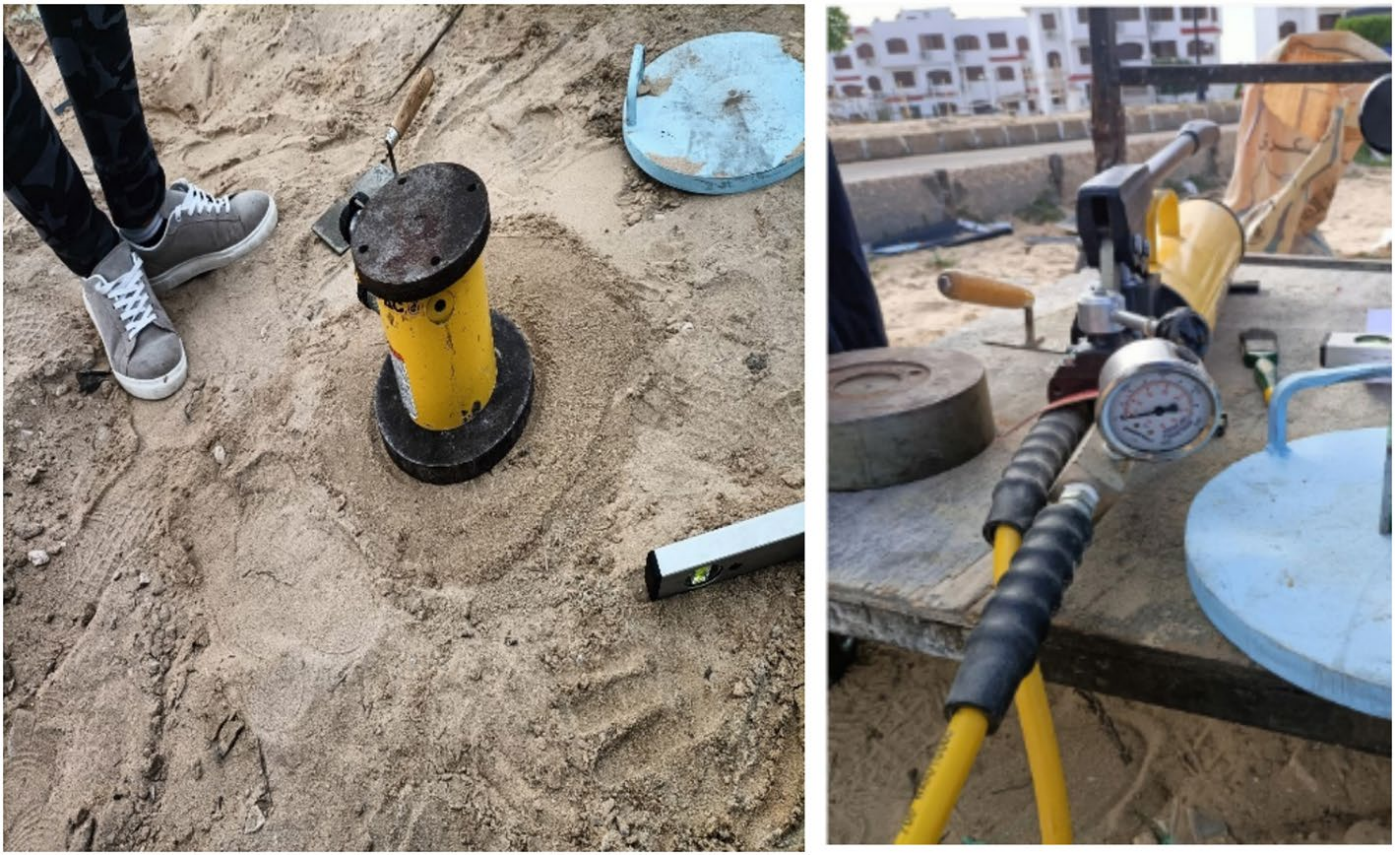
Loading jack and pump.

The testing load was applied using a 50 tons hydraulic jack located at the top of the tested column as shown in Fig. 7.

#### Test of group (1)

**Fig. 8:**
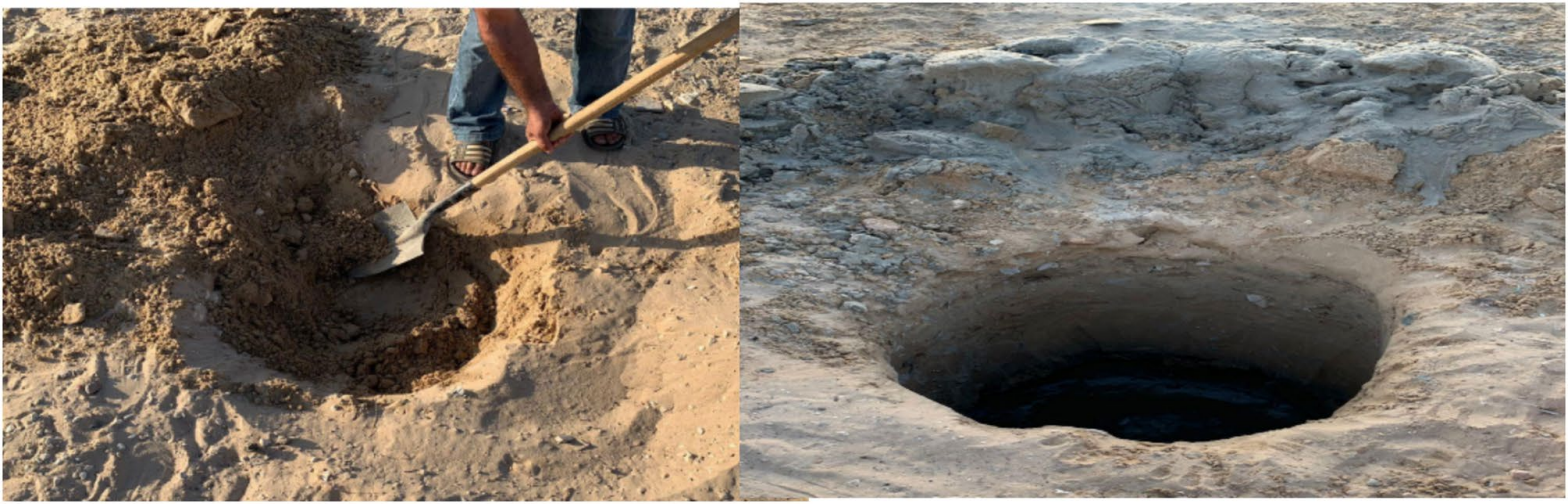
Work site and soil chamber and loading frame.

It was necessary to allocate a place for experiment. The site selected and soil chamber to implement the test in Sinai university in North Sinai, after that, A hole was drilled 1.0m deep and 0.6 m diameter, as shown in Fig. 8.

**Fig. 9:**
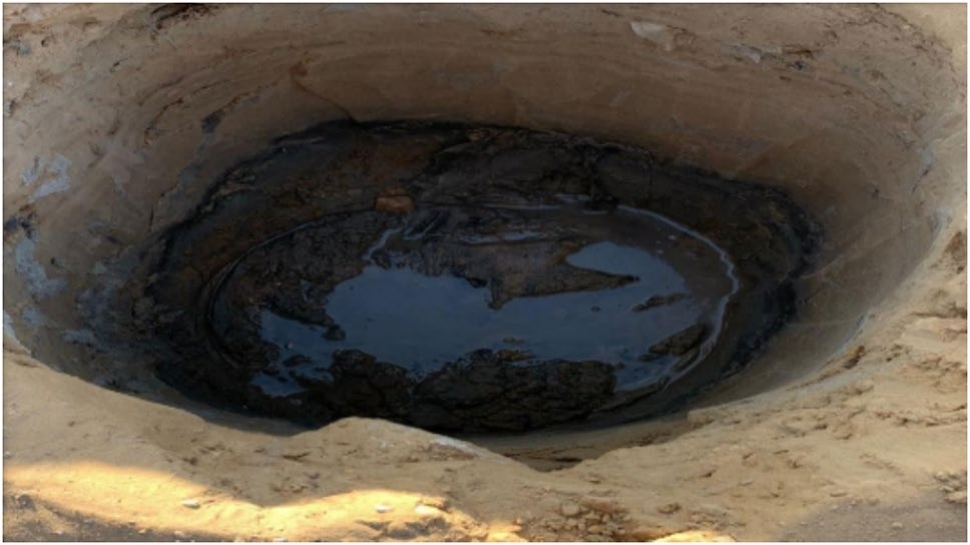
Ground water in the site.

When the hole was drilled, groundwater was found, as shown in Fig. 9

**Fig. 10:**
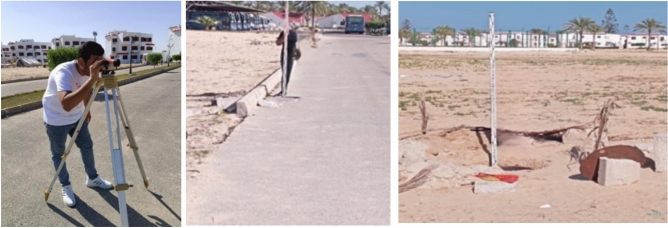
Determination the Ground water level.

**Fig. 11:**
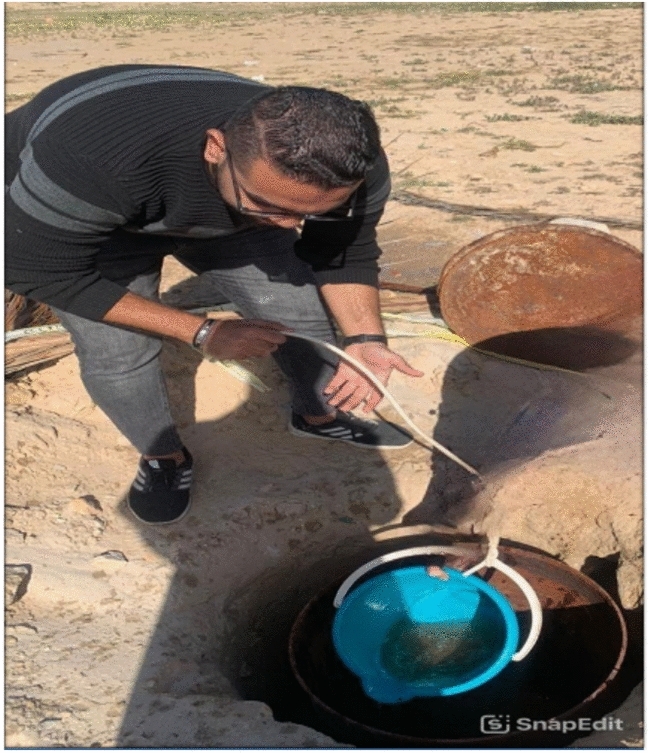
Dewatering the under-ground water.

After the presence of ground water, it was measured using a level instrument to monitor it in case its level rises, as shown in Fig. 10 After taking the ground water level, it became clear that the water had increased, and the water was drained as shown in Fig. 11.

**Fig. 12:**
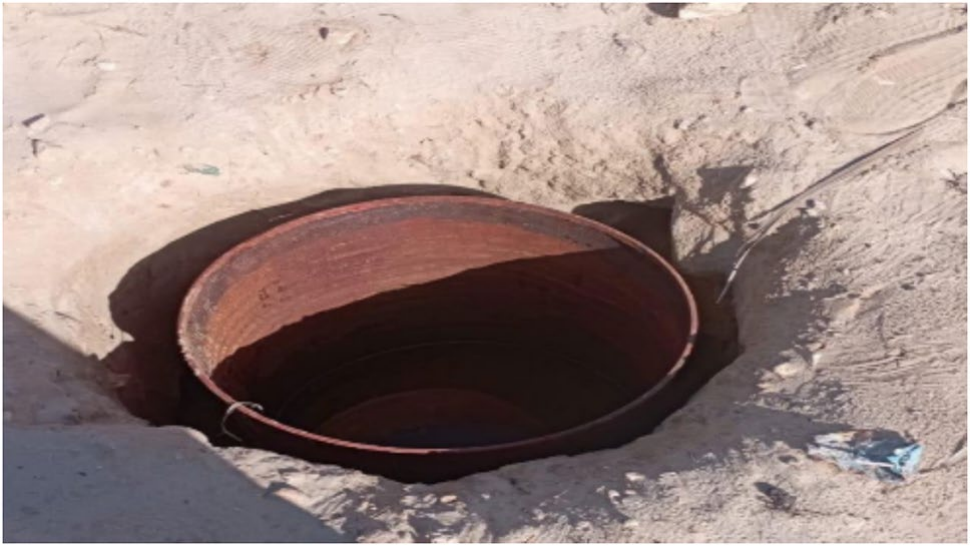
Soil Chamber.

After the drilling of the hole, the sides bonded and the most appropriate solution was by barrel, as shown in Fig. 12.

**Fig. 13:**
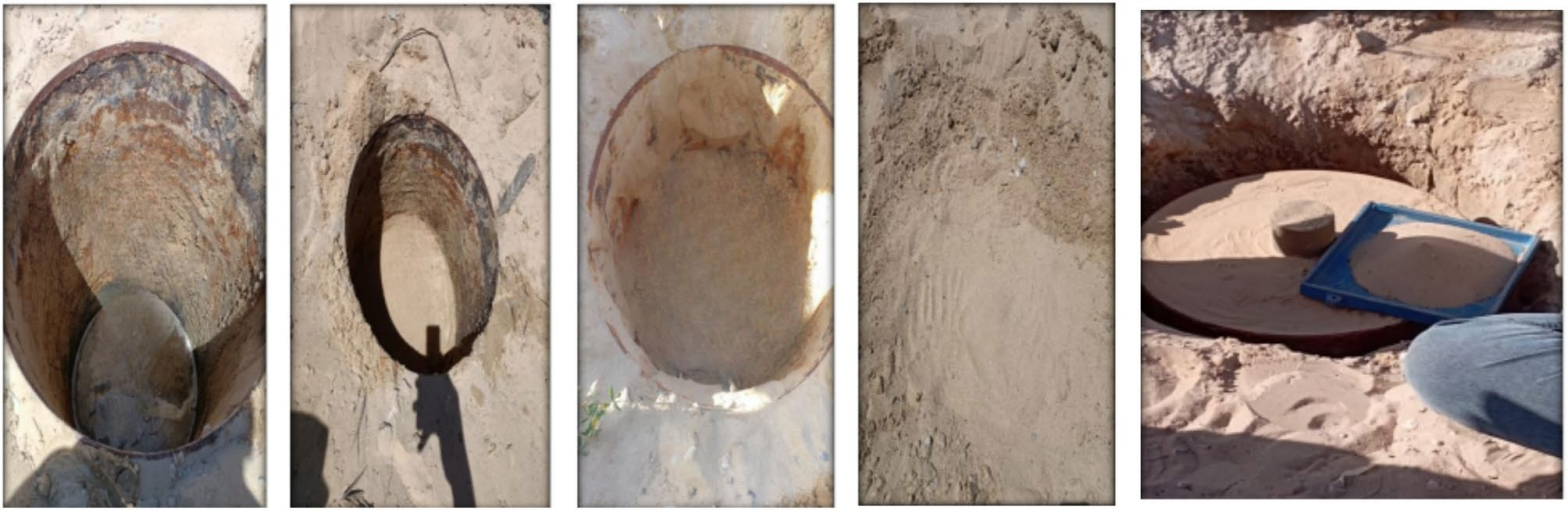
Compact the soil and sand cone test.

The soil was tested to determine its bearing capacity without a modification to the soil, as.

drilling output in the soil chamber using a rain technique to have water content of maximum dry density and sand cone test was performed to check on four layers as shown in Fig. 13.

#### Test of group (2)

**Fig. 14:**
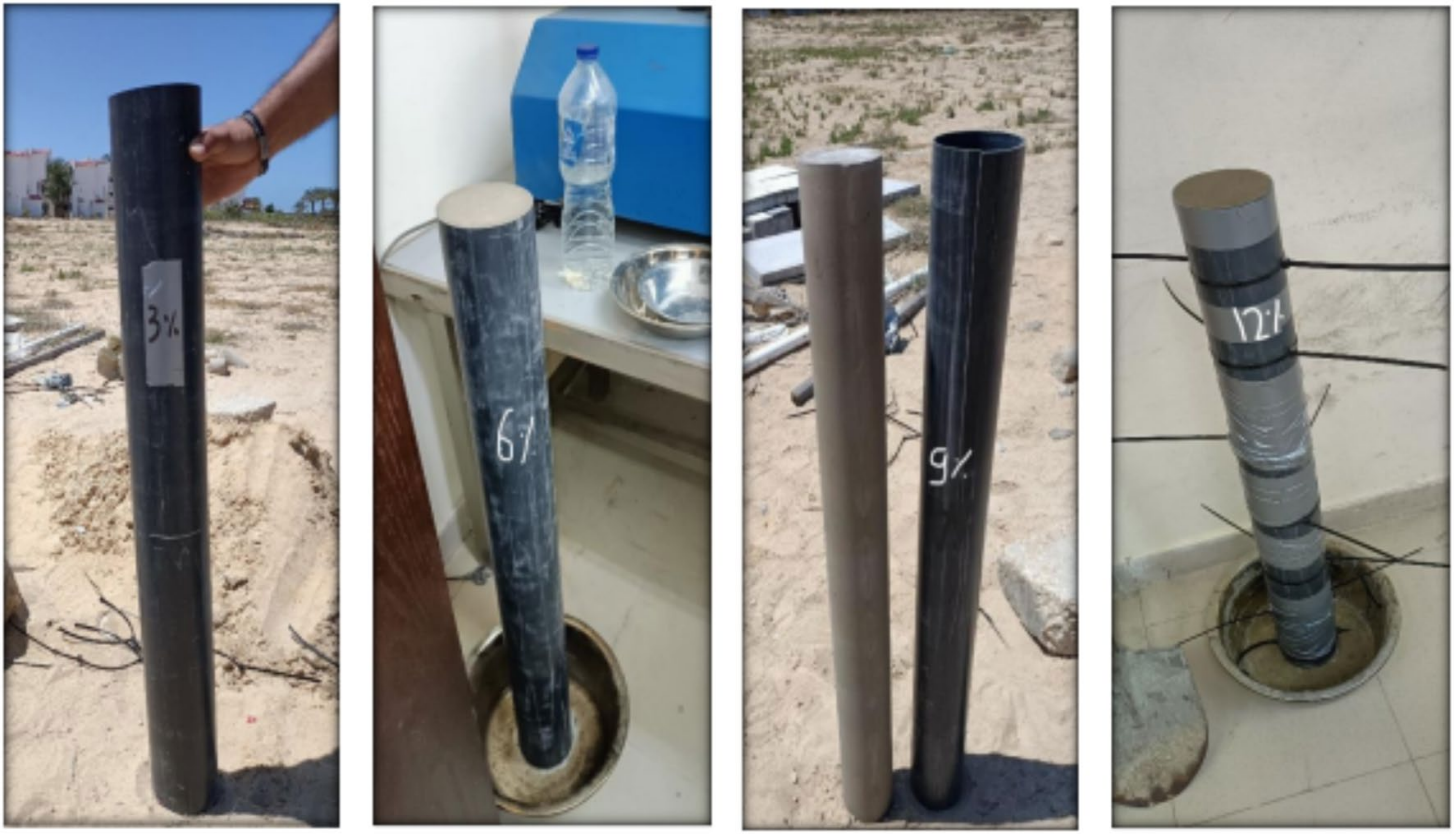
The cement soil column with various percentage of cement (3,6,9 and 12%).

**Fig. 15:**
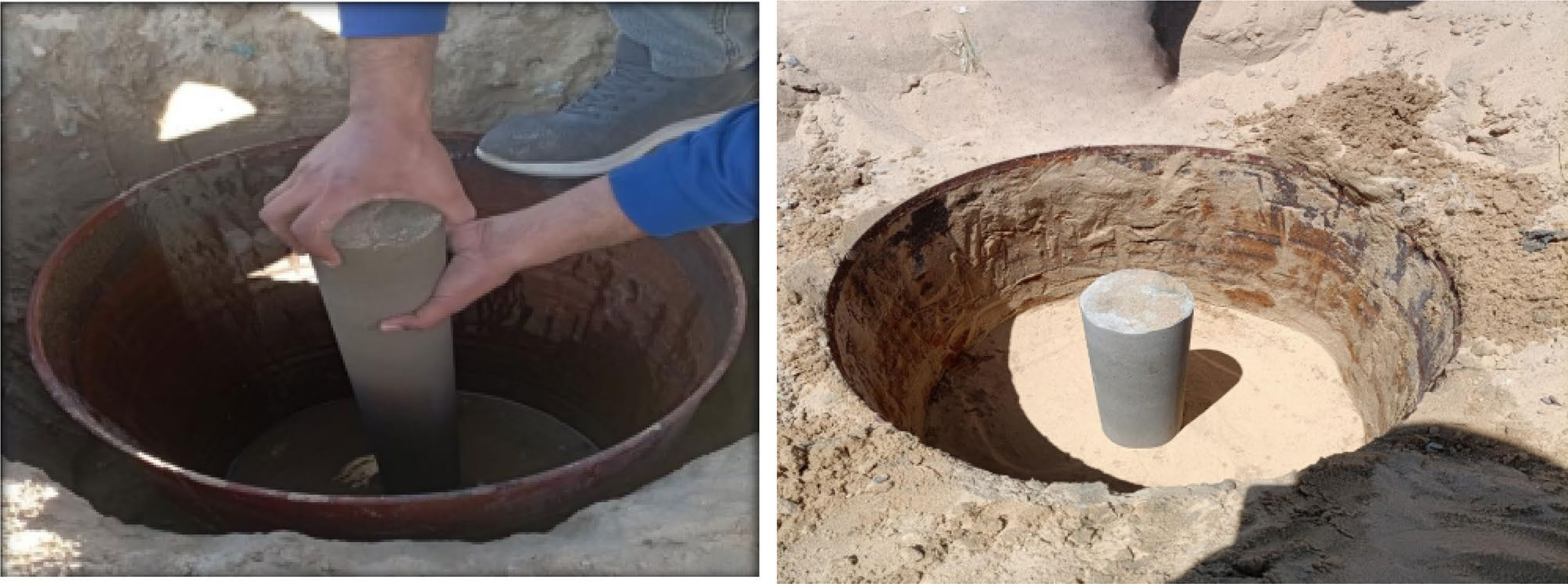
Placing cement soil column in the soil chamber.

**Fig. 16:**
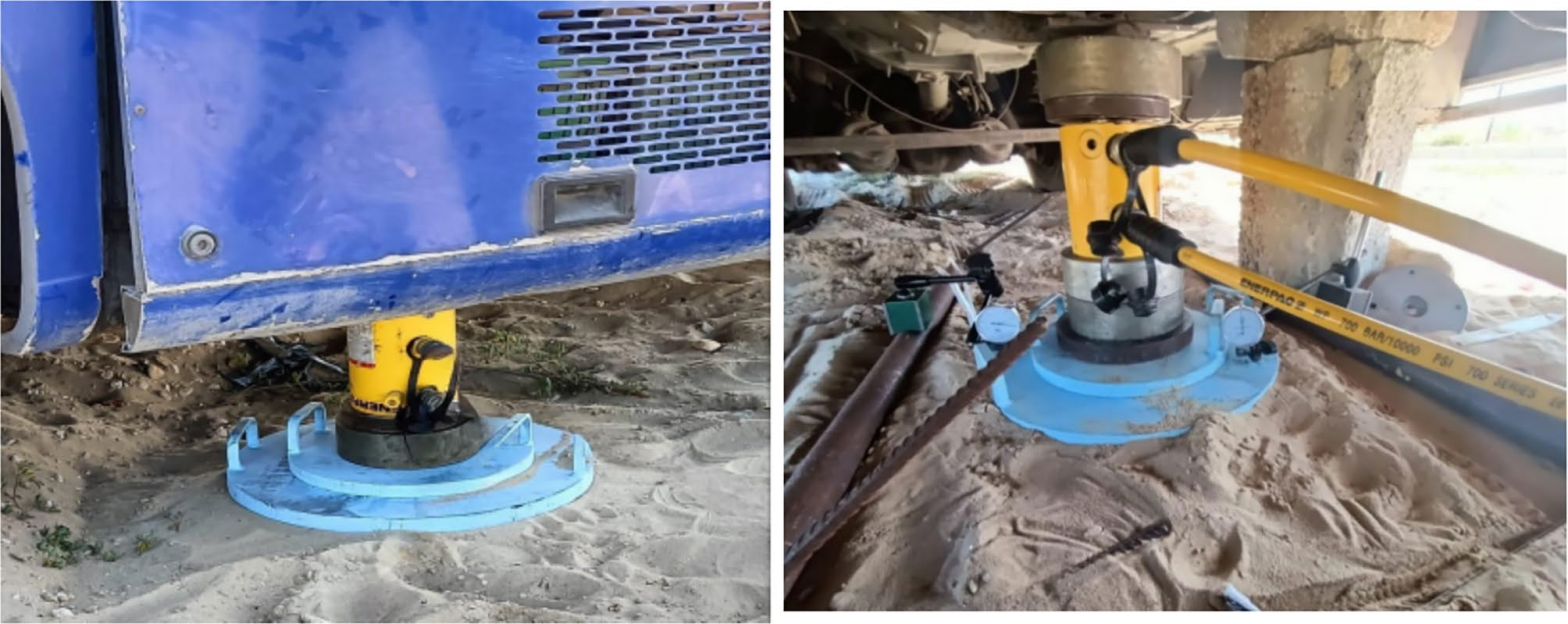
The cement soil column testing.

The cement columns were embedded in the compacted layers of a very fine-sandy soil with traces of clay in El-Arish region such that the total embedment depth of the cement column was 1.0 m after filling the soil chamber with 25 cm of sand using manual compactor (hammer). However, the vertical displacements of cement column were measured by two dial gauges with accuracy of 0.001 cm and the percentage of cement was variable from (3,6,9 and 12%) as shown in Figs. 14, 15, 16.

## Experimental result

**Fig. 17:**
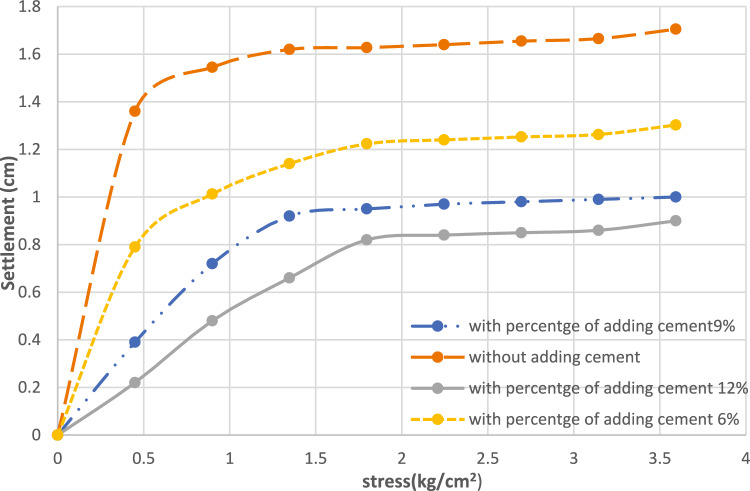
Load–settlement response of cement column for various cement contents (Lcc = 1.0 m, D = 0.1 m at df = 0.0).

**Fig. 18:**
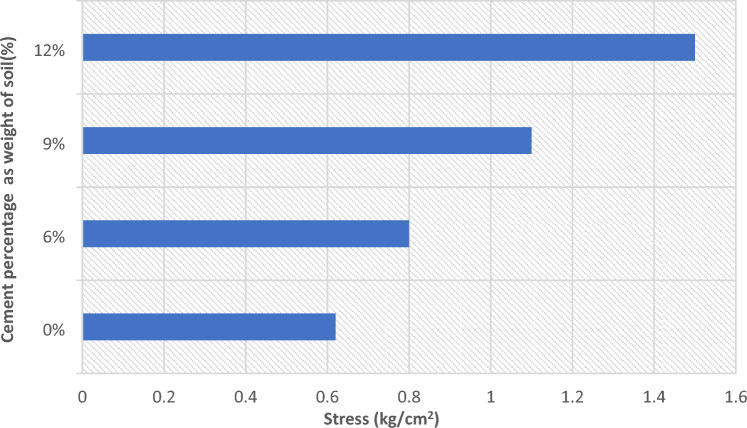
Comparison between allowable bearing capacity for different percentage of cement for cement column (Lcc = 1.0 m, D = 0.1 m).

Figure 17 shows the relation between stress and settlement at various percentage of cement for cement column and Fig. 18 shows the Comparison between allowable bearing capacity for different percentage of cement for cement column (L_cc_ = 1.0 m, D = 0.1 m).

It was found that the load–settlement behavior presented in Fig. 17 shows a clear enhancement in soil stiffness as the percentage of added cement increases. The untreated soil (without cement) settlement reaching approximately 1.65 cm at the surface at a stress level of 4 kg/cm^2^, which indicates high compressibility and limited load-carrying capacity. When cement is added, the settlement decreases considerably. At 6% cement content, the settlement curve shifts downward, reflecting partial improvement in soil behavior; however, the reduction is not yet optimal, as final settlement remains around 1.3 cm under the same stress level. At 9% cement content, a more improvement was observed, where settlement is reduced to about 1.0 cm. This indicates that the cement’s interaction with the soil has reached a level capable of forming a more continuous cementitious matrix, thereby increasing the soil’s resistance to deformation.

The best performance is achieved at 12% cement content, where settlement does not exceed 0.9 cm. This confirms that increasing the cement content leads to increased bonding between soil particles, resulting in greater rigidity and improved load-bearing capacity. Overall, the results indicate that cement contents above 6% produce significant improvements, and that cement contents between 9 and 12% achieve the greatest reduction in sludge. This finding is consistent with previous studies on soil–cement stabilization, which indicate that higher cement ratios generate denser and stronger soil composites.

## Analysis of finite element

### Single cement column

#### Model verification

To ensure the accuracy of the finite element model, a critical step in this study was the verification of the finite element model against experimental results. This was done to ensure that the numerical simulations accurately replicate the real physical behavior of the single cement-soil columns. The model was verified for columns with 6%, 9%, and 12% cement content to illustrate its reliability across the range of stabilization levels investigated. The material parameters for the cement-soil columns were carefully selected and calibrated based on the experimental results and established empirical relationships.

**Fig. 19:**
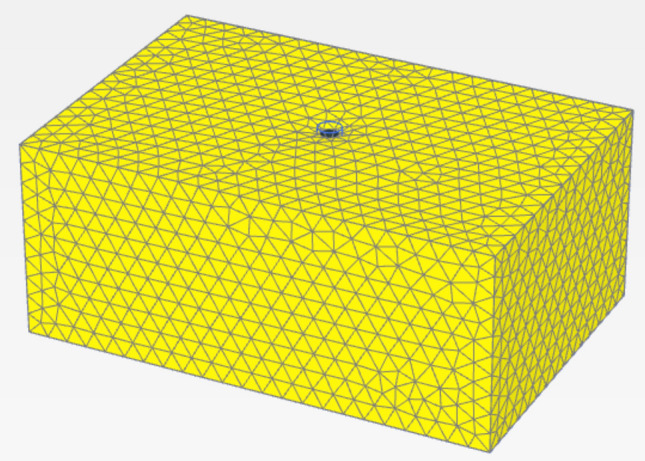
Deformed mesh of cement column (Lcc = 1.0 m, D = 0.1 m and cement percentage = 6%).

**Fig. 20:**
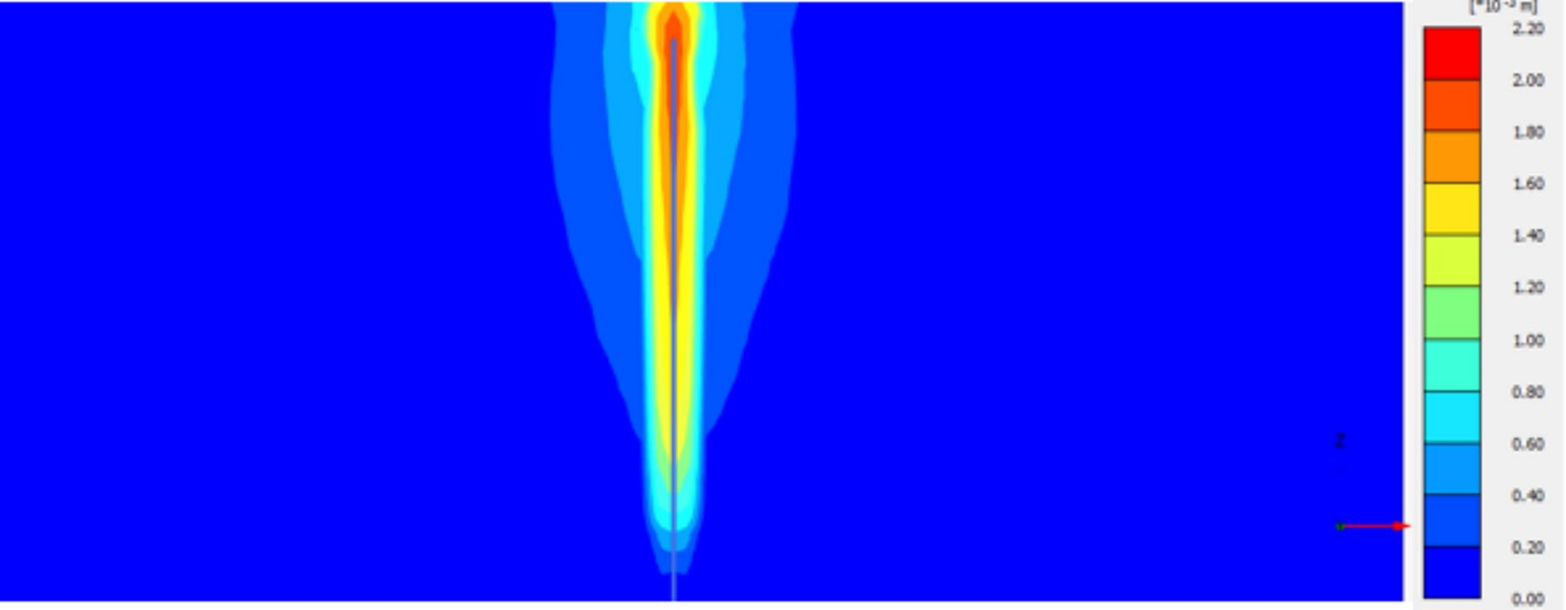
Vertical displacement (Settlement) as shading for cement column (Lcc = 1.0 m, D = 0.1 m and cement percentage = 6%).

**Fig. 21:**
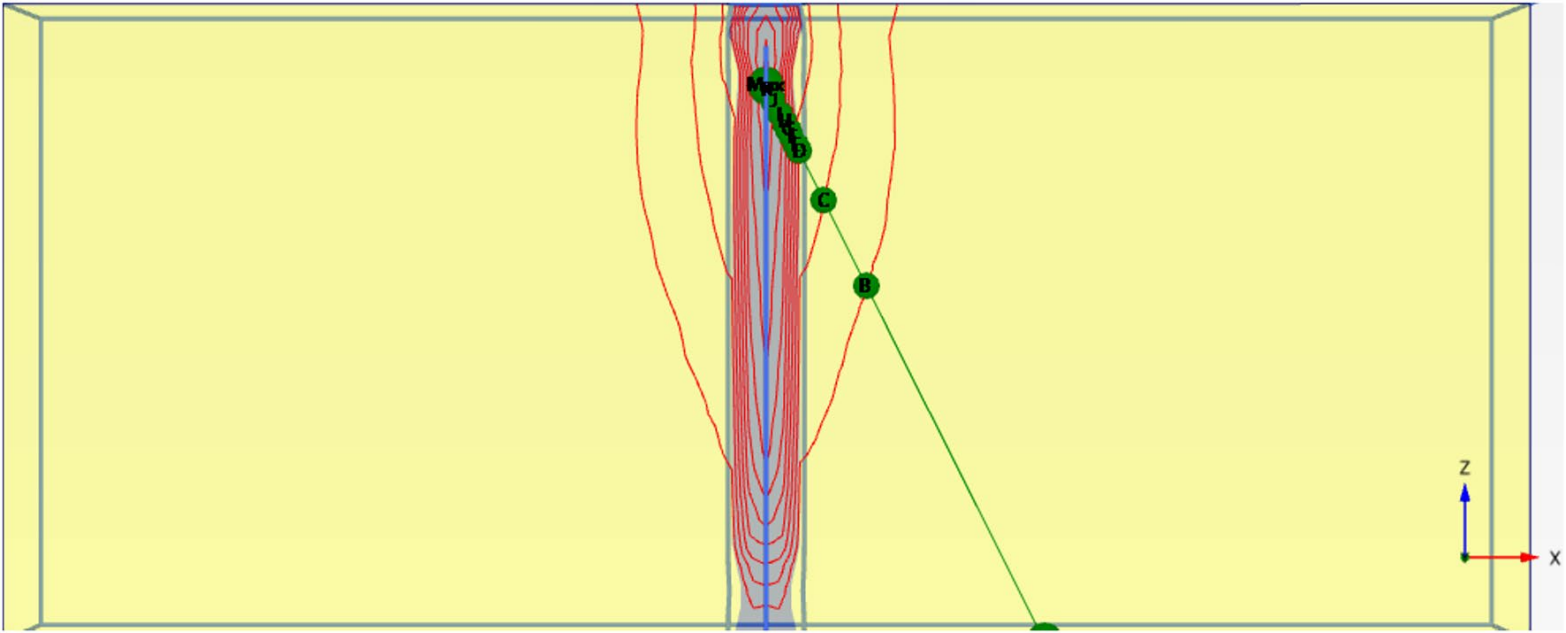
Vertical displacement (Settlement) as contour for cement column (Lcc = 1.0 m, D = 0.1 m and cement percentage = 6%).

**Fig. 22:**
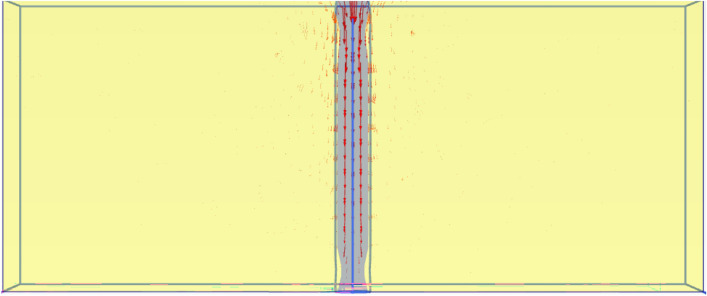
Vertical displacement (Settlement) as arrow for cement column (Lcc = 1.0 m, D = 0.1 m and cement percentage = 6%).

The calibrated parameters for the cement-soil columns are presented in Table 3 and Figs. 19, 20, 21, 22. The primary variables adjusted during calibration were the cohesion (c’) and Young’s Modulus (E), as these most directly influence the shear strength and stiffness observed in the load-settlement curves. These parameters were scaled based on the unconfined compressive strength of each mixture, following established empirical relationships^11^.

**Table 3:** Calibrated material parameters for cement-soil columns.

Parameter	6%CS column	9% CS column	12% CS column	Unit
Material Model	Mohr–Coulomb	Mohr–Coulomb	Mohr–Coulomb	-
Unit Weight, γ	16.2	16.2	16.2	kN/m^3^
Young’s Modulus, E	8,000	14,000	20,000	kPa
Poisson’s Ratio, ν	0.3	0.3	0.3	-
Friction Angle, φ	32	32	32	°
Effective Cohesion, c’	15	30	40	kPa
Dilatancy Angle, ψ	1	2	2	°
Interface Strength, R	1	1	1	-

**Fig. 23:**
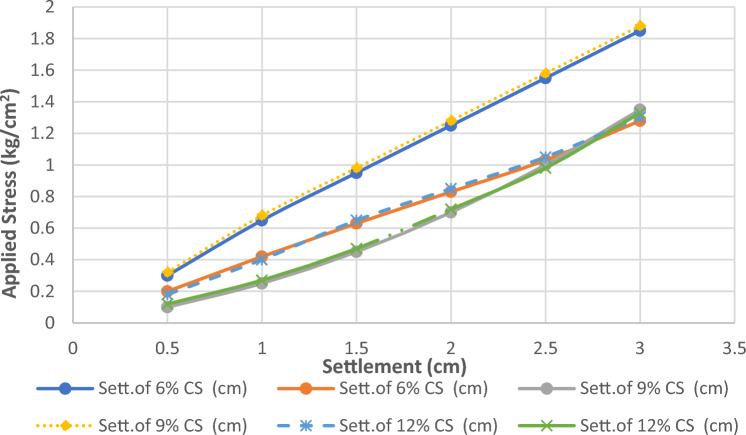
Comparison between Experimental and Numerical Settlement Values of cement columns with various percentage of cement.

The comparison between the experimental and numerical load-settlement behavior is presented quantitatively in Table 4 and graphically in Fig. 23. The results illustrate a close agreement for all three cement percentages. The model successfully captures the key behavioral trends: the significant compressibility of the 6% mix, the intermediate improvement of the 9% mix, and the high stiffness and low settlement of the 12% mix. The close correlation observed across all mixes validates the chosen Mohr–Coulomb constitutive model and the calibrated parameters. This successful verification provides high confidence in the results of the subsequent numerical parametric studies investigating group column configurations.

**Table 4:** Comparison of Experimental and Numerical Settlement Values.

Applied Stress (kg/cm^2^)	Sett. of 6% CS (cm)		Sett. of 9% CS (cm)		Sett. of 12% CS (cm)	
	Exp	FEM	Exp	FEM	Exp	FEM
0.5	0.30	0.20	0.10	0.32	0.18	0.12
1	0.65	0.42	0.25	0.68	0.4	0.27
1.5	0.95	0.63	0.45	0.98	0.65	0.47
2	1.25	0.83	0.70	1.28	0.85	0.72
2.5	1.55	1.03	1.00	1.58	1.05	0.98
3	1.85	1.28	1.35	1.88	1.30	1.33

### Group of cement column

**Fig. 24:**
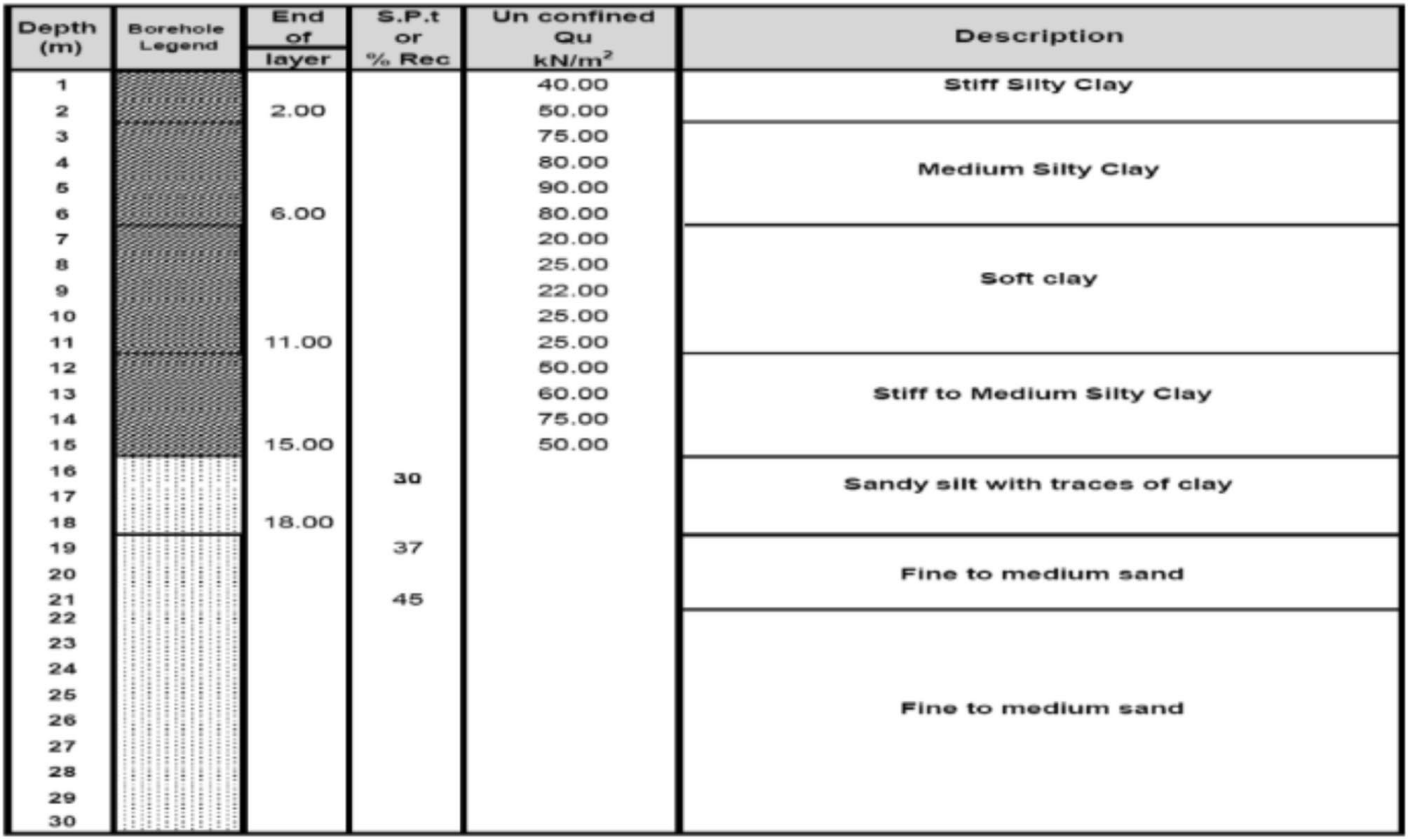
Borehole Log for Soil used Sesmeta, Beni-Suef Governorate, Egypt Project.

After the experimental program carried out theoretically and it was found a close agreement between experimental and theoretical results, a theoretical analysis have been done for a selected site (in governmental project in Semesta, Beni-suef, Governorate, Egypt. Figure 24 illustrates a borehole for the pervious site was chosen to be used in the analysis. The soil consists of six layers and simulated by a semi-infinite element isotropic homogenous elastic material. The properties of soil listed in Table 5. It is important to note that the analysis is based on a subsurface profile commencing at the ground surface.

**Table 5:** Properties for Soil Layers.

Parameters	Name	Stiff Silty Clay	Medium Silty Clay	Soft Clay	Stiff to Medium Silty Clay	Sandy Silt with Traces of Clay	Fine to Medium Sand	Unit
Material Model	-	Mohr–Coulomb	Mohr–Coulomb	Mohr–Coulomb	Mohr–Coulomb	Mohr–Coulomb	Mohr–Coulomb	-
Thickness	T	2	4	5	4	3	12	m
Young’s Modulus	Es	5000	4000	2000	4500	7500	15,000	kN/m^2^
Unit Weight	γ	16.65	16.35	15.65	16.55	17	17.5	kN/m^3^
Poisson’s Ratio	ν	0.25	0.3	0.3	0.3	0.3	0.25	-
Cohesion	c	50	40	12.5	25	25	0	kN/m^2^
Friction Angle	φ	0	0	0	25	25	30	°

#### Effect of numbers of cement columns

The first analysis is studying the effect of increasing the number of cement column (N = 1,2,3 and 4) cement column under fixed area of plate (A_p_ = 8.0 X 8.0) m and their diameters are fixed (D = 0.6 m) and constant length (Lcc = 34D) with percentage of cement = 12%. The material parameters for the cement-soil columns were calibrated to accurately replicate the experimental load-settlement behavior. The initial estimates for the parameters were based on empirical relationships with the measured Unconfined Compressive Strength of the 12% cement mixture, which was determined to be 80 kPa."

The cohesion (c’) was initially estimated as **q**_**u**_**/2.** Where q_u_ = (Maximum Load at Failure) / (Cross-Sectional Area of the Sample).

**Fig. 25:**
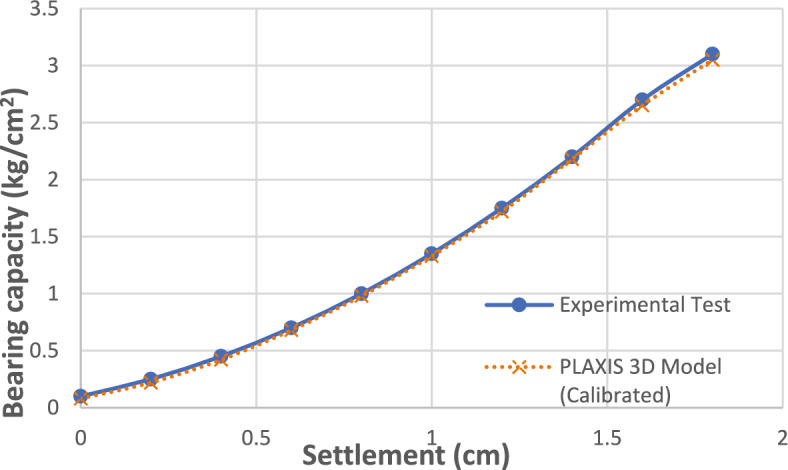
Validation of FEM Model for 12% Cement-Soil Column.

**Fig. 26:**
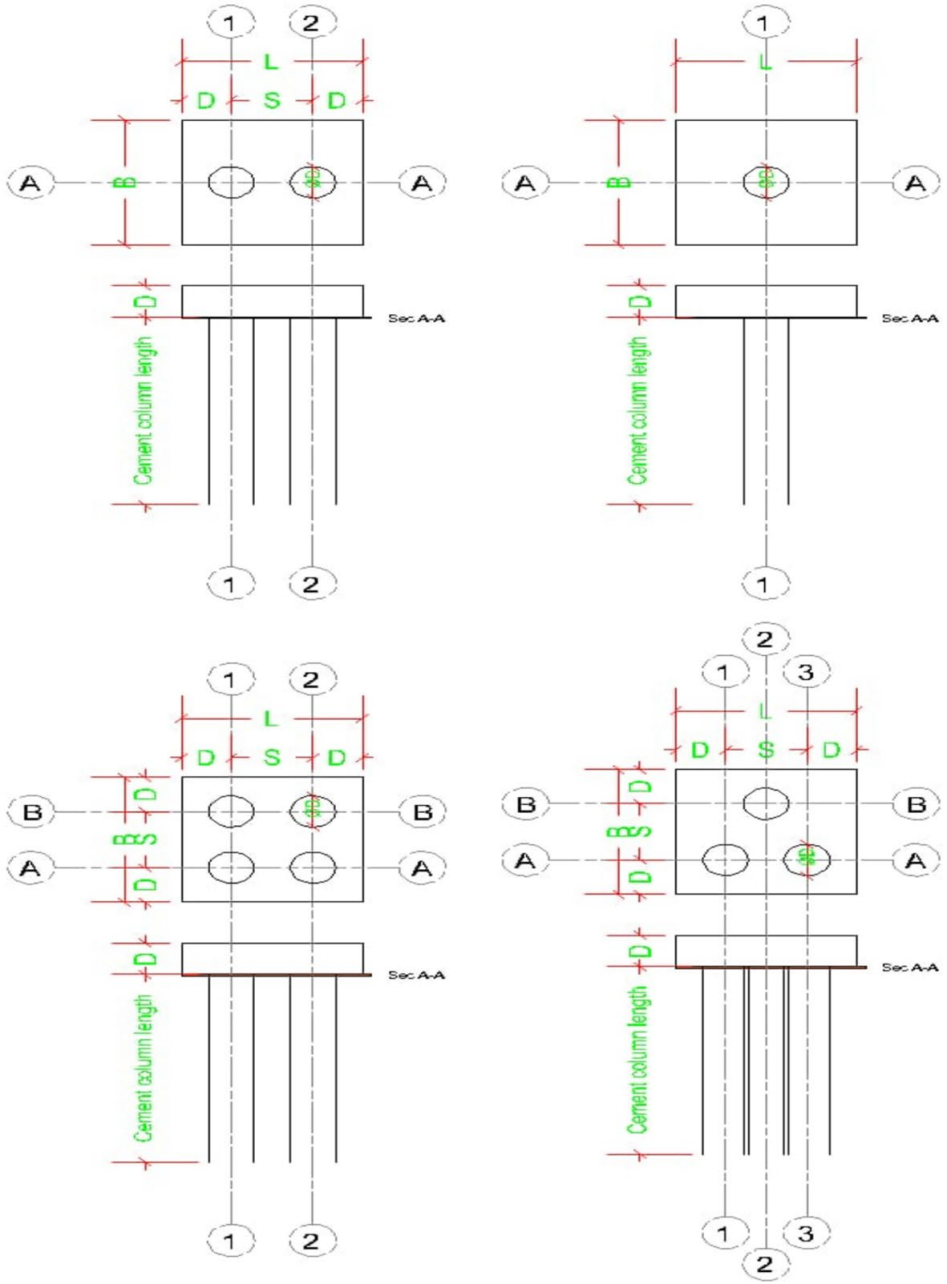
Models of various number of cement columns with in same area.

The Young’s Modulus (E) was initially estimated within the range of (150 to 250) * qu, a typical correlation for cemented sandy soils. These initial values were then iteratively adjusted in the PLAXIS 3D model until the numerical output for the single 12% cement column test showed a close match with the physical test results. The final calibrated parameters, listed in Table 5, were used for all subsequent numerical analyses. Figure 25 demonstrates the close agreement between the calibrated model and the experimental data, validating the selected parameters. The details and variation of these selected parameters are listed in Tables 6 and 7. And Figs. 25 and 26.

**Table 6:** Investigated cases of study.

No	Number of cements column	Cement column Diameter	Cement column Length
1	1	0.6	34D
2	2		
3	3		
4	4

**Table 7:** Properties for cement column.

Parameters	Cement column
Material model	Mohr-Columb
Material behavior	Undrained -A
Unit weight, unsaturated	16.2
Unit weight, saturated	16.2
Friction angle	32
Dilatancy angle	2
Effective cohesion	40
Horizontal permeability in X	5.66E-5
Horizontal permeability in Y	5.66E-5
Youngs modulus	20,000
Poisson ratio	0.3
Interface strength	1

##### Finite element model

**Fig. 27:**
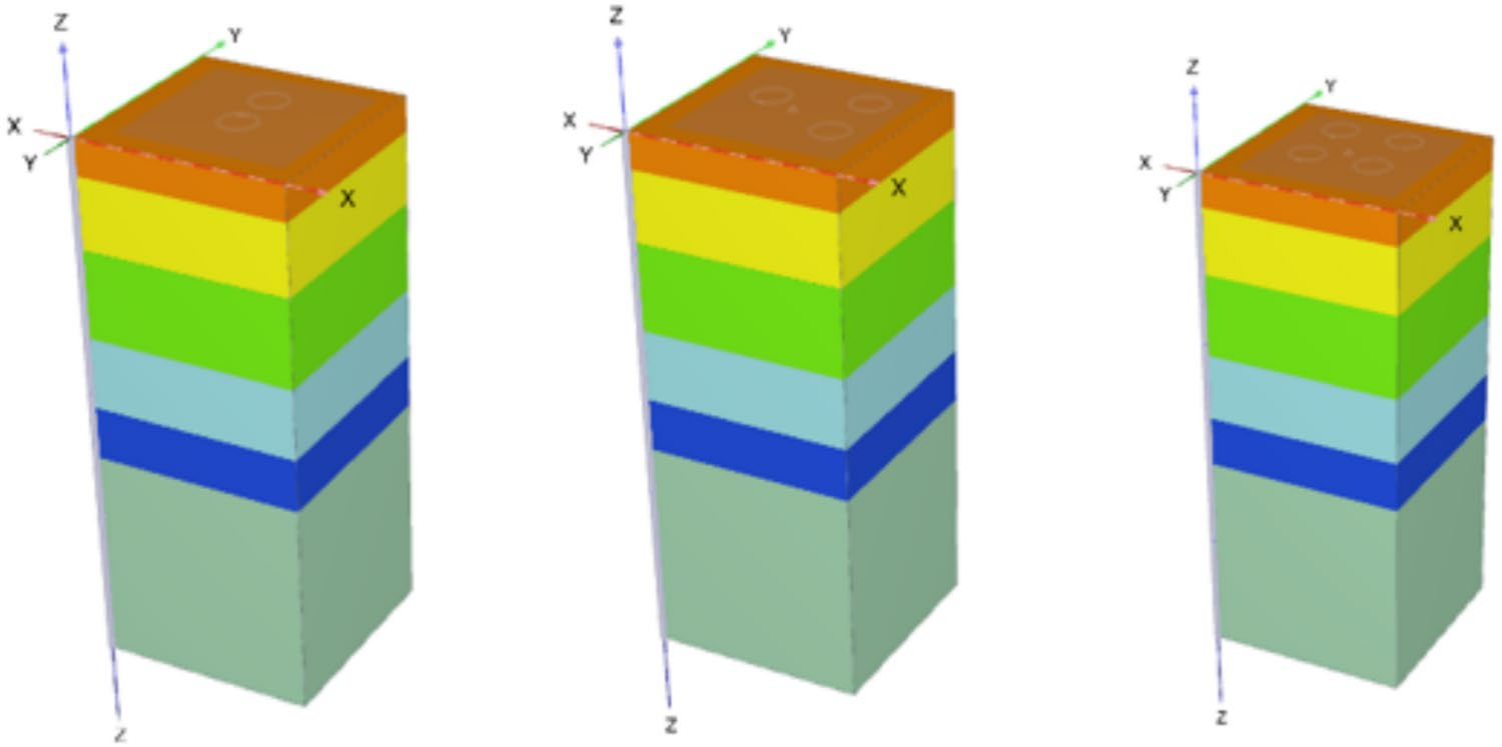
Plan 3-D For various number of Cement Columns (Lcc = 34d, Scc = 3d, D = 0.6 M).

**Fig. 28:**
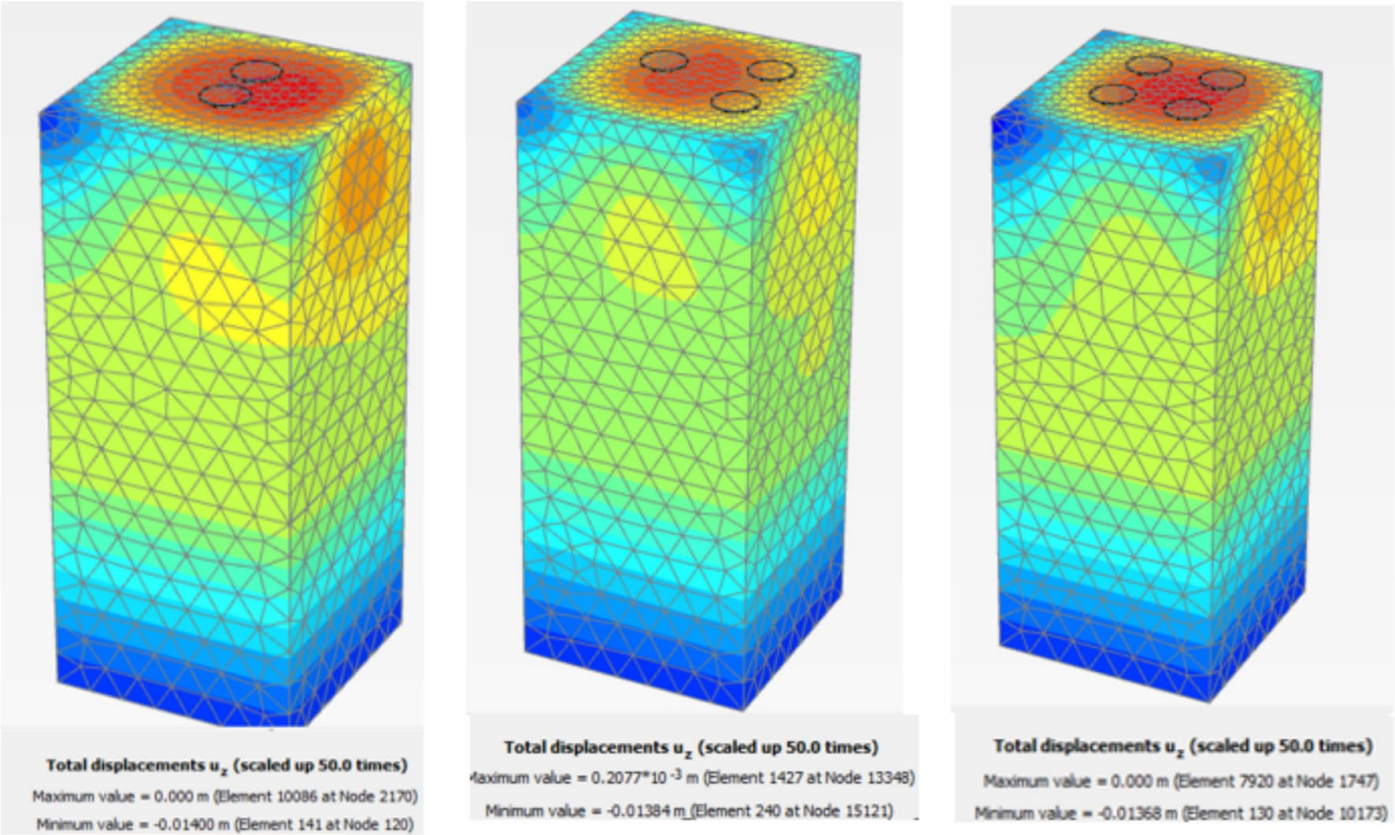
Vertical displacement as contour in Plan 3-D For various number of Cement Columns (Lcc = 34d, Scc = 3d, D = 0.6 M).

Figure 27 shows the plan in 3-D for a selected example for the model of (N = 2,3 and 4) cement column (L_p_ = 34D, D = 0.6 m S_cc_ = 3D). Figure 28 shows the vertical displacement for soil with various number of cement columns (N = 2,3 and 4) (L_p_ = 34D, D = 0.6 m S_cc_ = 3D at d_f_ = 0.0). It was found that increasing number of cement column in same area decrease the settlement of soil.

#### Effect of spacing between four cement columns

**Fig. 29:**
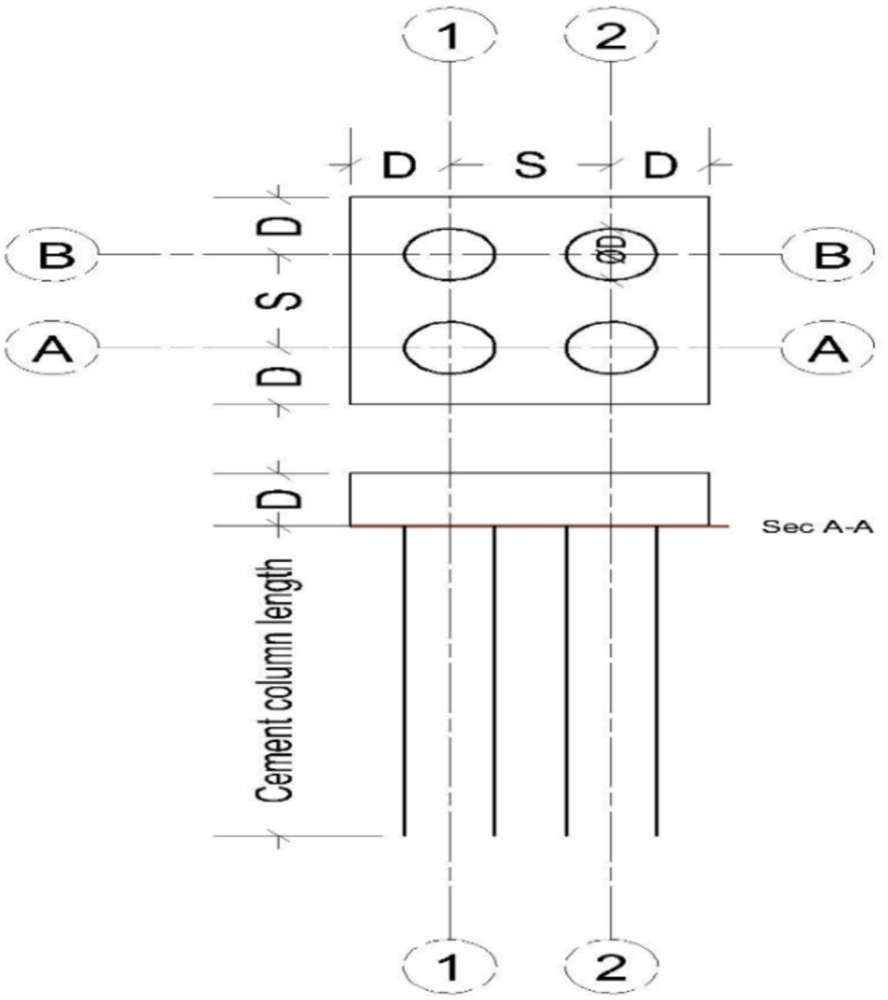
Cross section of four cement column model.

The Second analysis program consists of four cement column their diameters are fixed (D = 0.6 m) and the spacing between piles are various (Scc = 2D, 2.5D, 3D and 3.5D) and their length are constant (L_P_ = 34D) with percentage of cement = 12%. The Cement column subjected to vertical load. The details and variation of these selected parameters are listed in Fig. 29 and Tables 7, 8.

**Table 8:** Investigated cases of study.

No	Number of cements column	Cement column Diameter	Cement column Length	Cement column spacing
1	4	0.6	34D	2 D
2				2.5D
3				3D
4				3.5D

##### Finite element model

**Fig. 30:**
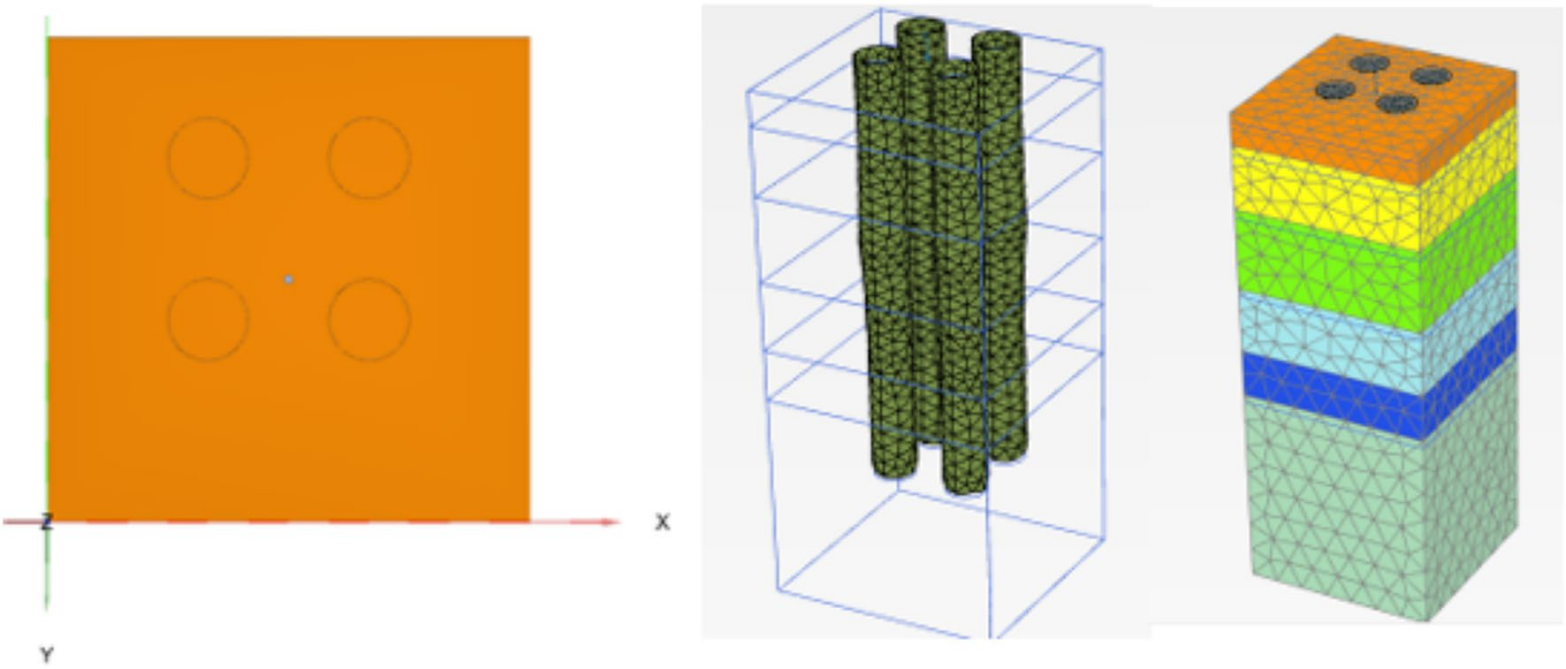
Plan In 2-D And 3-D For Four Cement Column (Lcc = 34d, Scc = 3D, D = 0.6 M).

Figures 30 shows the plan in 2-D and 3-D for a selected example for the model of Four cement column (Lp = 34D, D = 0.6 m Scc = 3D).

## Theoretical results

The theoretical results involve the following:

- i. Deformed mesh of the soil.
- ii. Settlement under Cement column.

### Finite element results

**Fig. 31:**
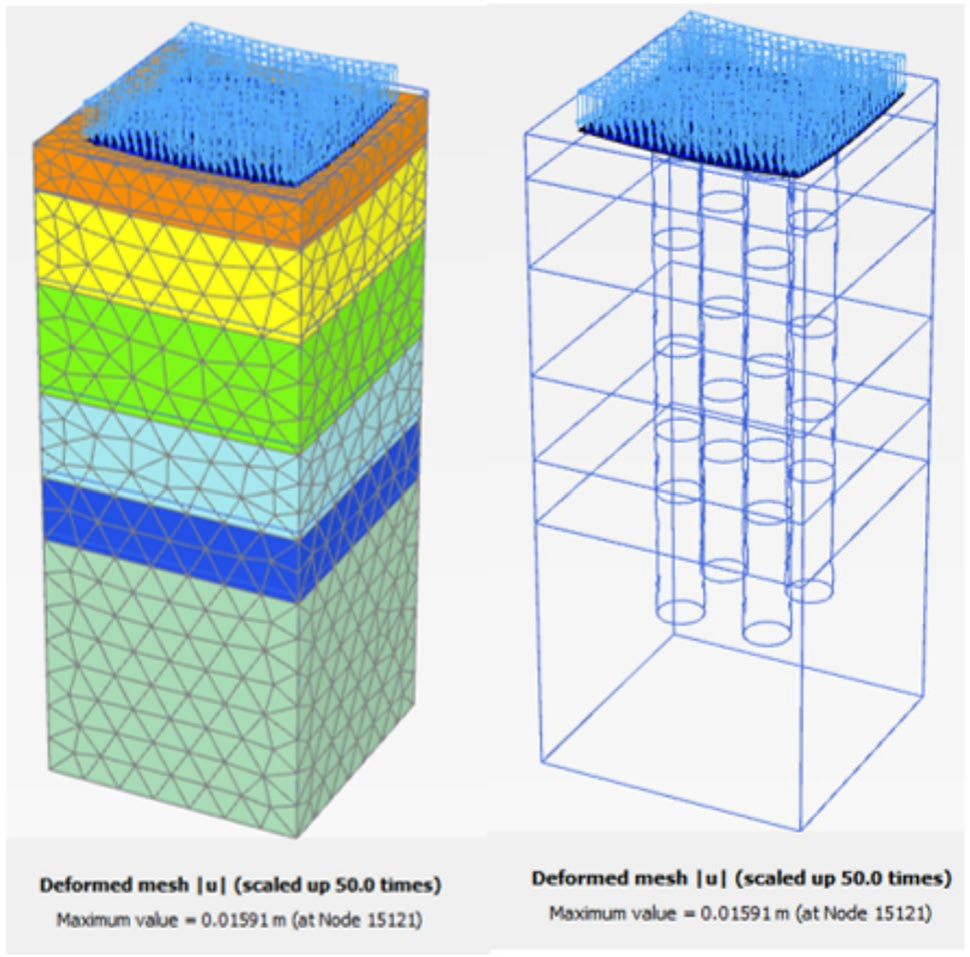
Deformed mesh for soil without cement column.

**Fig. 32:**
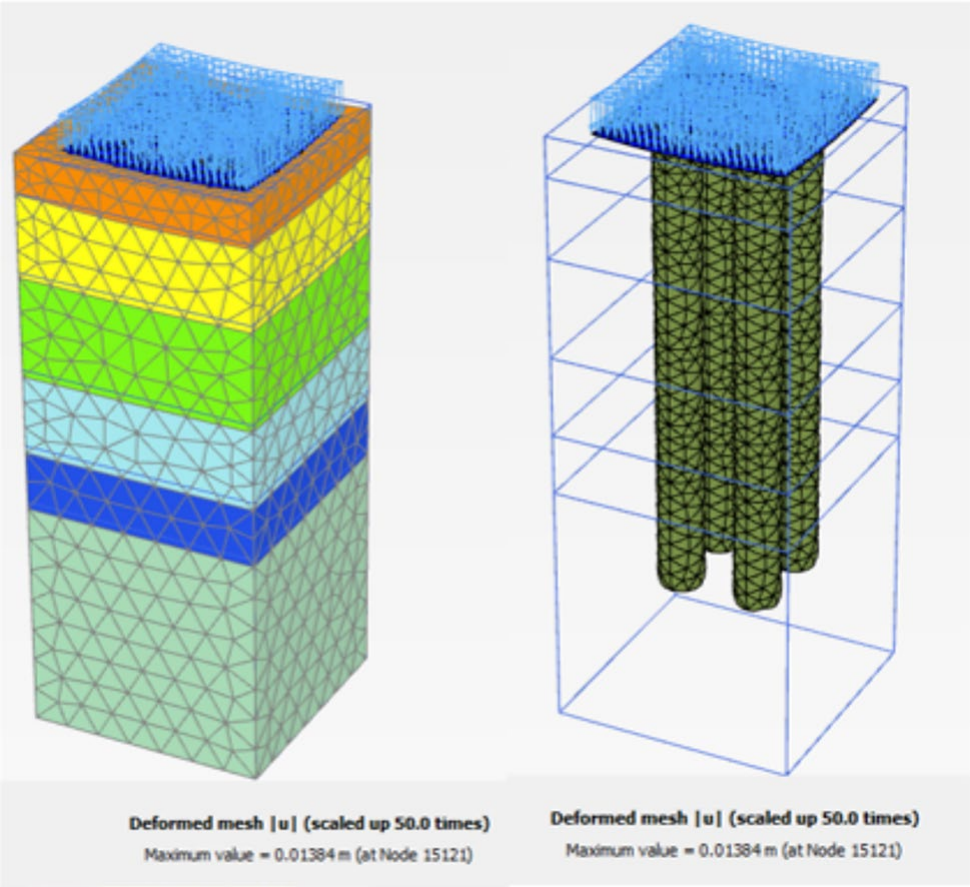
Deformed Mesh for Soil with Cement Column (Lcc = 34D, Scc = 2D, D = 0.6 m).

**Fig. 33:**
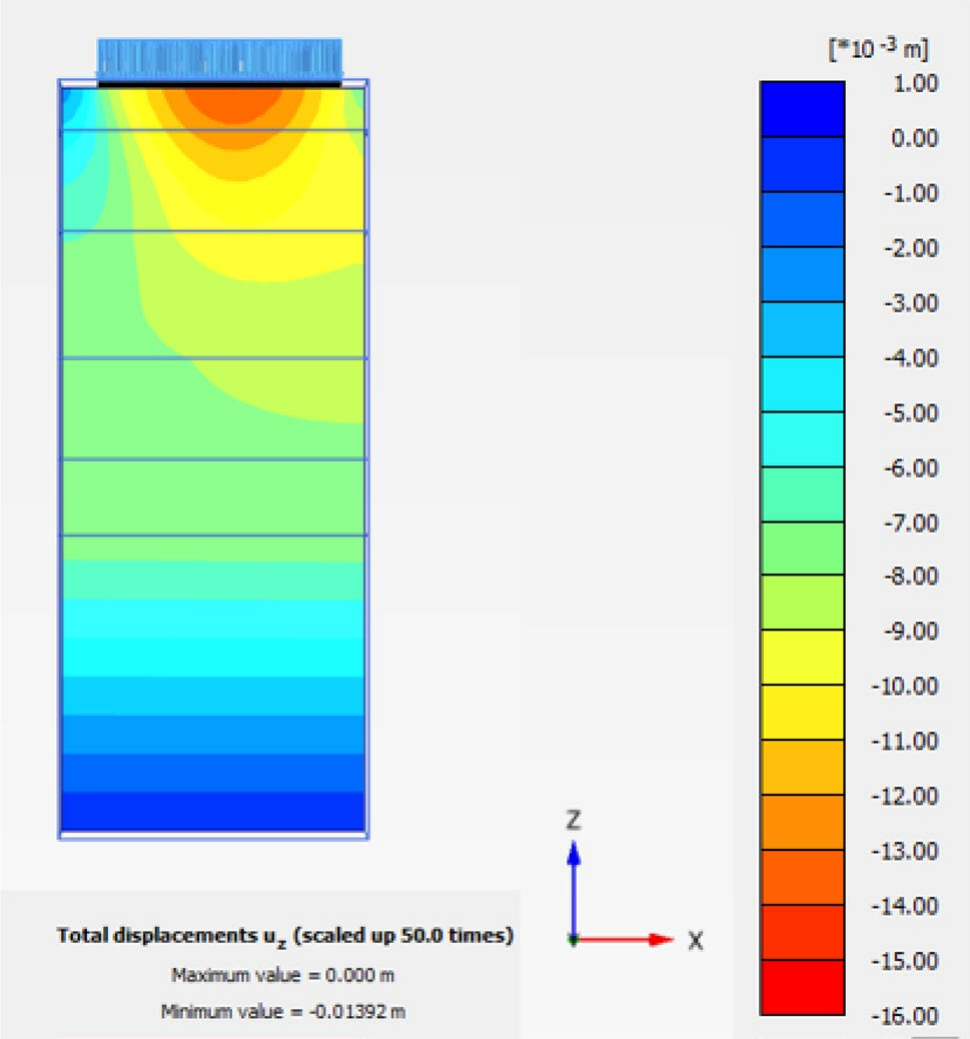
Vertical Displacement as Shading for Soil without Cement Column (Lcc = 34D, Scc = 2D, D = 0.6 m at d_f_ = 0.0).

**Fig. 34:**
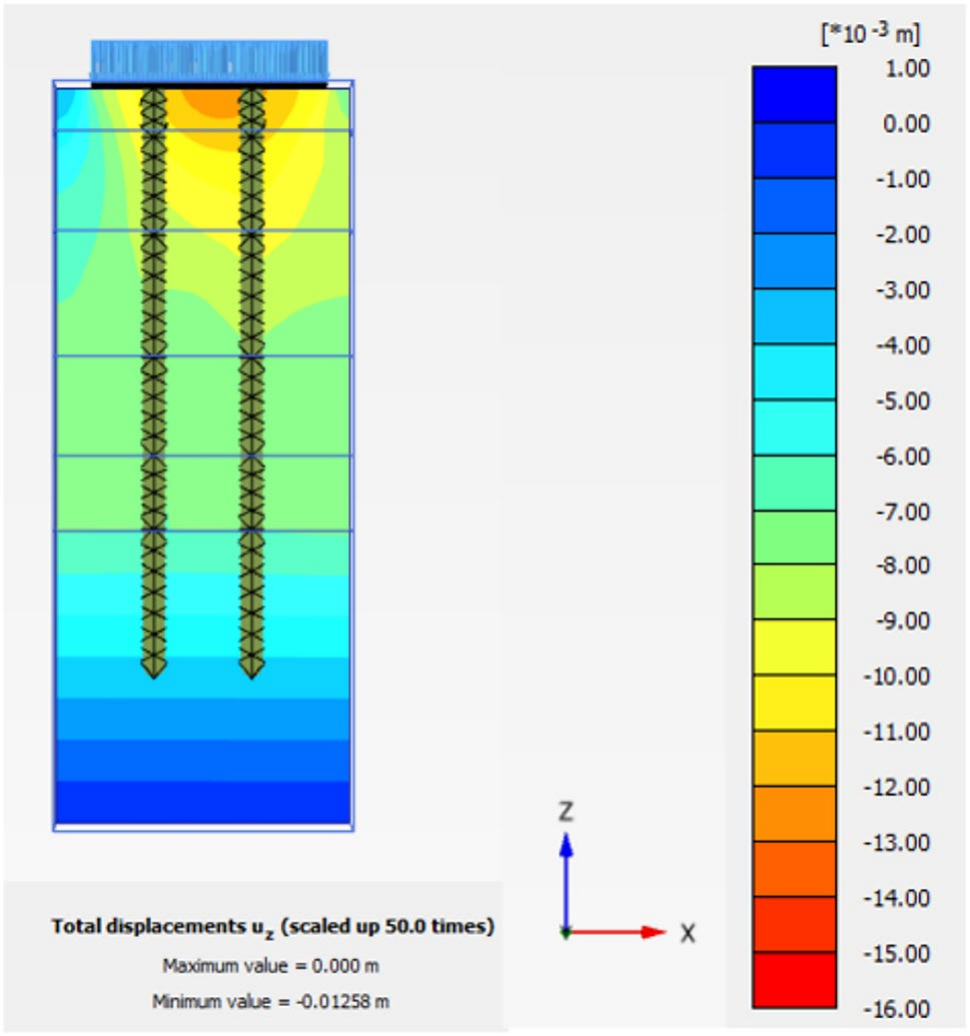
Vertical Displacement as Shading for Soil with Cement Column (Lcc = 34D, Scc = 2D, D = 0.6 m at d_f_ = 0.0).

**Fig. 35:**
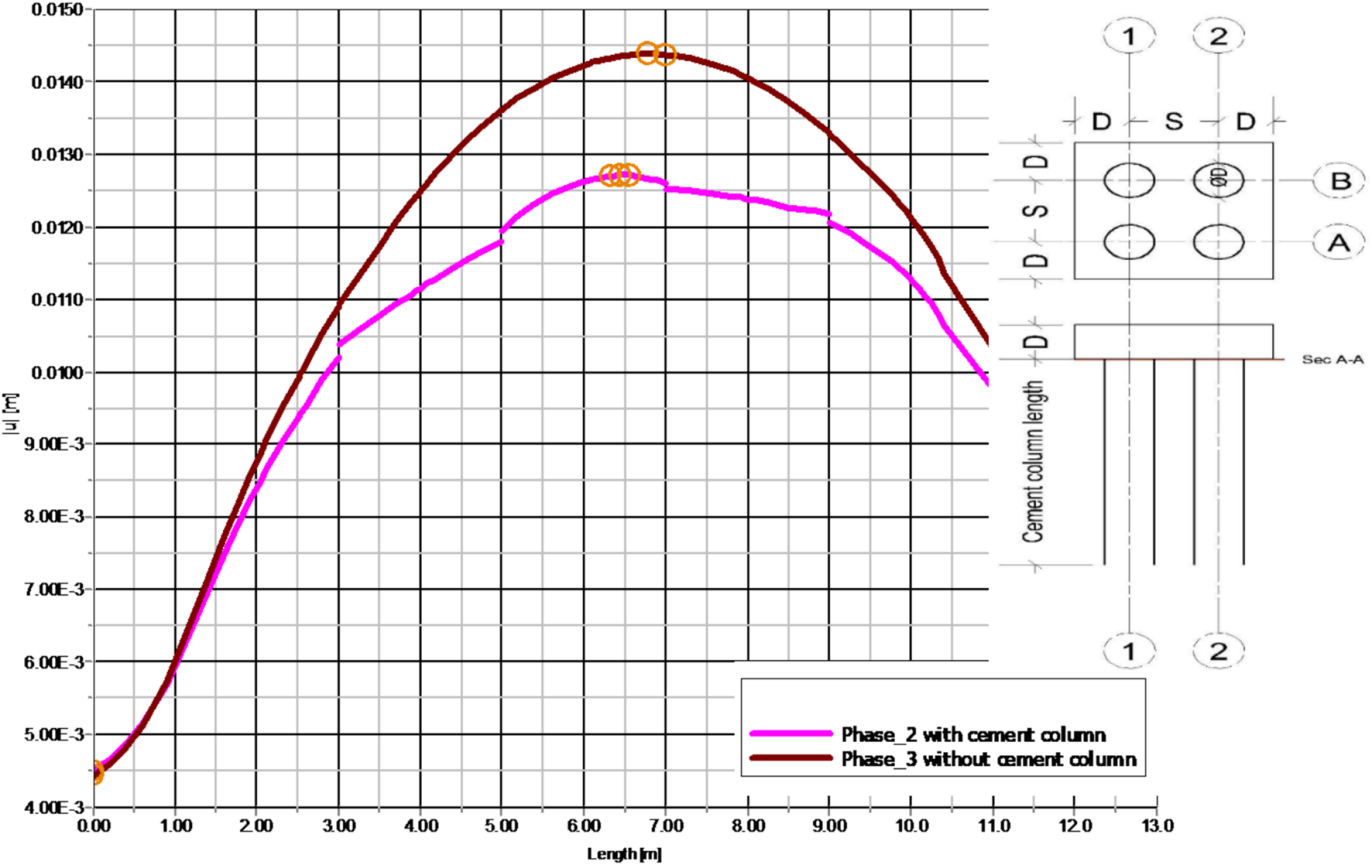
The relation between settlement for soil with and without cement column and distance along section (A-A) at (LP = 24D, SP = 3 D, D = 0.6 m).

**Fig. 36:**
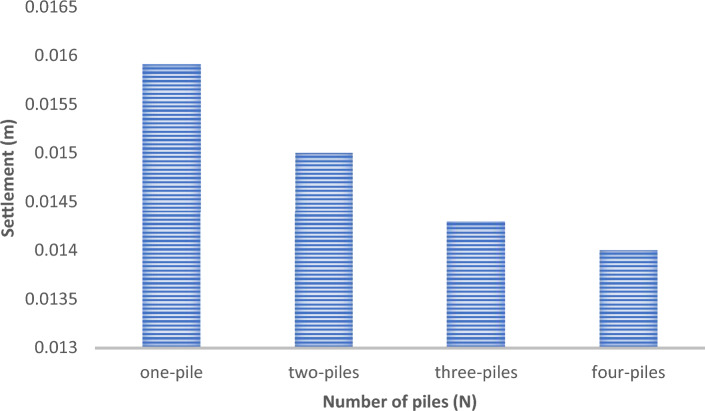
Comparison between Settlement for soil at different numbers of cement columns (L_cc_ = 24 D m, D = 0.6 m).

**Fig. 37:**
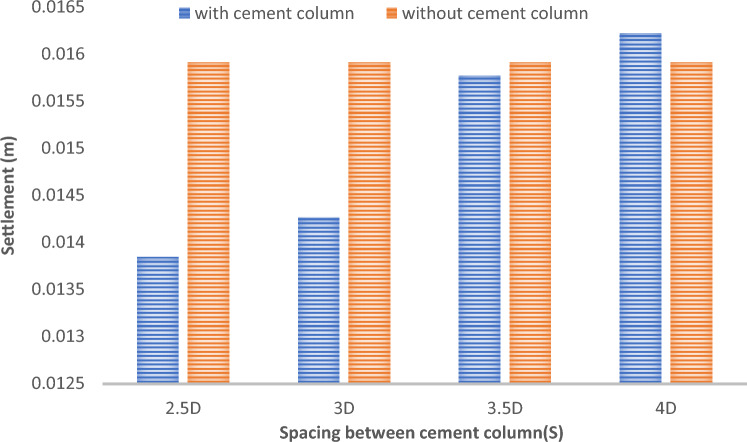
Comparison between Settlement for soil with cement column at different spacing and without cement column (Lcc = 24 D m, D = 0.6 m).

The obtained results of selected examples for different cases are shown in Figs. 31, 32, 33, 34, 35, 36, 37 as follows:

Figures 31 and 32 shows the deformed mesh for soil with and without four Cement columns subjected to raft load (Lcc = 34D, D = 0.6 m Scc = 2D). Figures 33 and 34 show the vertical displacement (settlement) as shading for soil with and without four Cement columns subjected to raft load (Lcc = 34D, D = 0.6 m Scc = 2D at d_f_ = 0.0). Figure 35 shows the relation between the settlement and distance along cross section (A-A). It was shown that the settlement of the soil without cement column is higher than the soil with cement column. Figure 36 shows the comparison between number of cement columns and settlement at same area. It was found that settlement increase by increasing number of cement columns. Figure 37 shows the comparison between spacing between cement column with and without cement column. It was found that settlement decrease by decreasing spacing between cement column.

## Conclusions

From the present study, the followings are concluded:

1. Increasing the cement ratio mixed in fine sandy soil leads to increase in shear strength.
2. Higher cement ratios generate denser and stronger soil composites.
3. The cement stabilization leads to decrease a vertical deformation with increasing the cement ratio.
4. Percentage of cement < 9% have no significant effect to enhance bearing capacity of soil
5. Cement ratio experiment of 3% has been failed.
6. Percentage of cement 9% creates a strong soil–cement composite, leading to a high reduction in settlement (up to 45%) and increase the bearing capacity and shear strength.
7. Settlement decreases by increasing number of cement column at same area but increases by increasing the spacing between cement column.
8. Settlement is most effectively controlled by using a group of columns with close spacing. as decreasing the spacing between columns and increasing their number within a group are the key design parameters for minimizing foundation settlement.

## Data Availability

Data is provided within the manuscript.
